# Investigating the Oxidative Potential and *In Vitro* Toxicity of Ambient Water-Soluble PM_10_ in an Eastern Mediterranean Site

**DOI:** 10.1021/acsestair.5c00085

**Published:** 2025-06-16

**Authors:** Zheng Fang, Alexandra Lai, Eli Windwer, Michal Pardo, Chunlin Li, Ajith Thenoor Chandran, Alexander Laskin, Yinon Rudich

**Affiliations:** † Department of Earth and Planetary Sciences, Weizmann Institute of Science, Rehovot 76100, Israel; ‡ College of Environmental Science and Engineering, Tongji University, Shanghai 200072, China; § Department of Chemistry, Purdue University, West Lafayette, Indiana 47907, United States

**Keywords:** particulate matter, biomass burning, dust event, oxidative potential, reactive oxygen species, cytotoxicity, EDTA

## Abstract

In this study, the acellular dithiothreitol (DTT) assay, the *in vitro* cellular DCFH-DA assay on human lung epithelial cells, and gene expression measurements were used to assess the toxicity of water-soluble (WS) PM_10_ relating to reactive oxygen species (ROS) in summer at an Eastern Mediterranean urban site. Large influences from anthropogenic sources on health risks were observed with acellular and cellular assays. Anthropogenic biomass burning (BB) and natural dust events increased human pulmonary exposure to the oxidative potential (OP_dose,T_) of WS-PM_10_ by 209 and 47%, respectively, compared to regular periods. OP_v_^DTT^ and ROS_v_ results were positively correlated in anthropogenic-dominant samples, while showed no significant correlation in the remaining samples. As a result, the BB and dust event had higher and lower levels of cellular ROS_v_ compared with the nonevent period, respectively. Source apportionment results suggest that specific organic contents (e.g., PAHs) had relatively low contents in samples less influenced by anthropogenic sources, possibly explaining the divergence in acellular and cellular results. Heavy metals were dominant contributors of OP_v_^DTT^ throughout the campaign, and a Chelex method is recommended over a EDTA method for quantification of their summed OP_v_^DTT^.

## Graphical abstract

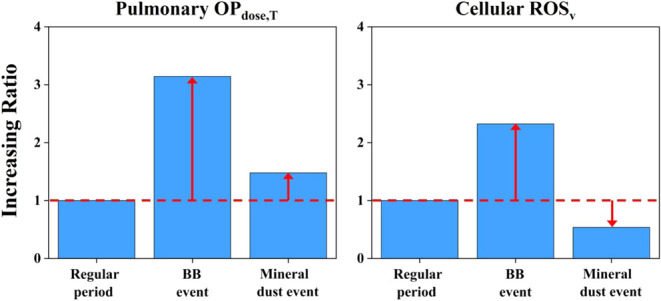

## 1 Introduction

Outdoor air pollution is a major threat to human health and is estimated to contribute to 4.2 million premature deaths annually worldwide.^1^ Numerous epidemiological studies have shown the effects of particulate matter (PM) on mortality and cardiovascular or respiratory morbidity.^2–5^ Some of these adverse health effects are associated with forming aerosol-induced reactive oxygen species (ROS), such as O_2_^•–^, ^•^OH, H_2_O_2_, and RO_2_^•^, which overwhelm the antioxidants in the cells and can cause harmful oxidative stress and inflammation. Previous studies have found that the intrinsic oxidative potential (OP) of PM varies substantially depending on its pollution sources, chemical components, and degree of oxidation.^6–8^ Additionally, different PM sizes may exhibit different OP levels and deposition rates in the human respiratory tract.^9^ These findings underscore the limitations of routine monitoring of PM mass concentration without physical and chemical information for understanding ambient PM’s health effects.

In the Eastern Mediterranean Region, dust storms have significant impacts on air quality; however, very few studies evaluating their acute health effects have been conducted in this region.^10^ Desert dust is the most abundant PM by mass in Earth’s atmosphere, and a significant portion of the Northern Hemisphere’s population is exposed to it.^11^ Climate change and unsustainable land use have been expanding the global dryland areas over the past decades, which is predicted to increase human exposure to desert dust in the coming years.^12^ Recent systematic reviews of epidemiological studies have linked desert dust exposure to various acute and long-term health outcomes.^13,14^ However, many studies have observed no increase in OP or *in vitro* cytotoxicity PM levels during dust events.^15–19^ This is because desert dust has relatively low intrinsic toxicity compared to PM from other sources.^3,16,17^ On the other side, anthropogenic pollutants adsorbed to dust particles can increase its toxicity.^19–21^

Lag BaOmer, a traditional bonfire festival that involves burning natural and industrial wood in Israel, exemplifies the air quality challenges posed by anthropogenic biomass burning (BB). BB is another significant source of atmospheric PM, particularly its organic components.^22,23^ The detrimental effects of anthropogenic BB on air quality have been documented.^24–28^ The BB-PM has a relatively high OP level compared to other sources,^29,30^ and its small particle sizes allow it to penetrate efficiently into the human respiratory tract.^31,32^ These characteristics have raised broad public concern about its adverse effects.^33^ Despite recent efforts by the Israeli Environmental Protection Ministry to discourage bonfires, PM levels in many areas still substantially increase during the festival.^34,35^

The dithiothreitol (DTT) assay has been widely used to evaluate PM-induced OP in laboratory and field studies.^36,37^ The DTT assay is cheaper and easier to use compared to *in vitro* and *in vivo* experiments. DTT contains two sulfhydryl groups and acts as an electron donor when it reacts with ROS species. This process mimics how sulfhydryl-containing antioxidants, such as glutathione, are consumed by ROS species in the human body.^38^ It has been reported that the acellular OP does not consider biological processes within cells, and its translation into cellular ROS can be dependent on the specific composition of PM.^39–41^ Given that the DTT assay has also been used to evaluate health risks of ambient PM in nearby regions affected by desert dust,^15,18,31^ it is warranted to investigate the relationship between OP^DTT^ and cellular ROS of PM in this region. Recent studies used ethylenediaminetetraacetic acid (EDTA) to chelate transition metals, attributing the difference in measured OP^DTT^ with and without EDTA to the metals’ contribution to the OP.^5,42,43^ Besides, EDTA was also used to stabilize the light-absorbing product 2-nitro-5-thiobenzoic acid (TNB) for minutes or hours.^44–46^ However, with EDTA’s multiple applications in the DTT assay, the possible artifacts introduced by it have not been systematically evaluated.

A two-week PM_10_ sampling campaign was conducted in central Israel in May of 2023 to evaluate the health risks associated with different pollution sources. Size-segregated PM samples were collected during a dust storm event, a BB event, and typical nonevent periods. In this study, OP^DTT^ and cellular ROS, two health-related endpoints of WS-PM_10_, were linked to pollution sources using the positive matrix factorization (PMF) receptor model. As marker species for anthropogenic and mineral dust sources, the contribution of HMs to OP^DTT^ was quantified with two different methods. The correlation between OP^DTT^ and cellular ROS behaved differently according to the influence of anthropogenic sources, and the BB event and desert dust event were chosen as two opposite cases to investigate such a relationship. Our findings highlight the severity with which WS-PM_10_ induces oxidative stress during the BB event. Inappropriate usage of EDTA can lead to a biased interpretation of the OP^DTT^ results, and a Chelex ion exchange treatment is suggested instead.

## 2 Materials and Methods

### 2.1 Bulk and Size-Segregated PM_10_ Sampling

Ambient PM_10_ samples were collected using a high-volume sampler (HiVol 3000, Ecotech) on the rooftop (15 m above ground) of a building at the Weizmann Institute of Science, Rehovot, Israel (N31°54′26.3″, E34°48′38.3″). Rehovot is a typical urban area in the Eastern Mediterranean region with population density of approximately 6500 km^–2^. Previous records from the collocated Surface PARTiculate mAtter Network (SPARTAN) continuous ambient sampling station showed that PM_10_ at this site has a large mineral dust contribution.^47,48^ In total, 25 quartz filter (Whatman QM-A) samples were collected from May 2 to May 15, 2023, representing the beginning of the hot and dry summer in a Mediterranean climate. The daytime samples were collected from approximately 9:00 to 21:00, and the nighttime samples were collected from approximately 21:00 to 9:00, and their detailed information is listed in Table S1. An extensive nationwide bonfire event began at sunset on May 8 and ended by sunrise the next day. Time resolution for the PM_10_ sampling over the festival event was set to 6 h, and two nighttime samples collected on May 8 (labeled 0508N1 and 0508N2) were characterized by relatively high biomass burning marker species (Figure S1). During this period, the wind predominantly came from the east (Figure S5). Efforts to pinpoint the exact locations of fire sources were not made, as multiple burning activities occurred on a community or family scale and were collectively considered a non-point pollution source. One sample with high mass loading of mineral dust was collected during the daytime on May 5 (labeled 0505D), which featured the highest PM_10_ concentration (219.8 ± 11.0 μg m^–3^) and the lowest PM_1_/PM_10_ ratio (0.06 ± 0.01) during the entire period.^18,49^

In addition to bulk PM_10_ sampling, seven size-resolved PM sample sets were collected using a 5-stage high-volume cascade impactor (TE-235, Tisch Environmental) installed in a high-volume PM_10_ sampler (TE-6070, Tisch Environmental). The 5 stages have D50 cut sizes of 7.2, 3.0, 1.5, 0.95, and 0.49 μm, yielding six size fractions. The smallest fraction (<0.49 μm) was collected on an 8 × 10″ filter (Whatman QM-A), while the larger fractions were deposited on slotted filters (TE-235-QZ). The sampling periods for the size-resolved PM samples covered the BB and mineral dust events and were kept simultaneous with their corresponding bulk PM_10_ samples (Table S1). All filters were prebaked at 500 °C for 5 h before sampling and stored at −20 °C afterward.

### 2.2 Chemical Speciation on WS-PM_10_

Ambient PM_10_ concentration data were obtained from the Rehovot Air Monitoring station, which is part of the Israeli Ministry of Environmental Protection network. The station is approximately 1 km from the sampling site and uses a continuous ambient particulate monitor (FH62C14, Thermo Fisher Scientific) to report PM_10_ concentration at 5 min intervals. A high-resolution time-of-flight aerosol mass spectrometer (HR-TOF-AMS, Aerodyne) was also deployed, and the fractions of *m*/*z* 44 (*f*_44_) and *m*/*z* 60 (*f*_60_) were used as tracers for secondary PM and BB-PM, respectively.^50^ Six punches (each 1.13 cm^2^) of each PM_10_ filter were extracted with vortex shaking (Vortex-Genie 2, Scientific Industries) in 11 mL of pure water for 3 h with a rotation frequency of 3200 rpm, and then the insoluble components were removed with a syringe filter (0.45 μm pore size, Millex). The concentrations of WS organic carbon (WSOC) were measured by a total organic carbon analyzer (TOC-VCPH, Shimadzu). Concentrations of three inorganic anions (sulfate, nitrate, and chloride) were measured by ion chromatography (ICS-3000, Dionex). The WSOC concentrations and the OM/OC ratios inferred from the AMS records were used to estimate the concentrations of WS organic matter (WSOM). To determine concentrations of the WS metals, water extracts were acidified with HNO_3_ (1% volume fraction) to keep metals suspended, and then they were analyzed using an inductively coupled plasma mass spectrometry (ICP-MS, Agilent 7700). In total, 16 metal species were analyzed: sodium (Na), magnesium (Mg), aluminum (Al), potassium (K), calcium (Ca), vanadium (V), manganese (Mn), iron (Fe), cobalt (Co), nickel (Ni), copper (Cu), zinc (Zn), arsenic (As), cadmium (Cd), Barium (Ba), and lead (Pb). Mass loadings of size-segregated PM_10_ filters were determined by an analytical balance (124i-1S, Sartorius Entris). The filter mass before and after sampling was measured for 3 times each.

### 2.3 OP and ROS Assays

For OP and ROS assays, all PM_10_ samples were extracted to achieve a standardized effective concentration of 0.1 g L^–1^ to eliminate concentration-based variability.^51,52^ At this concentration, OP and ROS for different samples were well differentiated, and ROS measurements on A549 cells were not significantly affected by cell death during cultivation (Table S2). For the cellular toxicological measurements, a comprehensive comparison of toxicity across samples would ideally include testing multiple concentrations to establish a dose–response relationship.^40,53–55^ However, this was not feasible in the current study due to limited sample quantities.

OP was measured by a dithiothreitol (DTT) assay, following procedures described in previous studies.^39,56,57^ Water was used as the solvent for extraction, and syringe filters were used to remove insoluble compounds. All PM_10_ and size-segregated PM samples were analyzed. Briefly, the assay involved a reaction between 100 μM DTT and 0.1 g L^–1^ PM under physiological conditions (pH = 7.4 and 37 °C). At 5 min intervals, aliquots were withdrawn from the solution and mixed with an excess of 5,5′-dithiobis­(2-nitrobenzoic acid), which reacts with residual DTT to produce TNB. The absorbance of TNB was measured by UV–vis spectroscopy (USB 650, Ocean Optics) at 412 nm immediately. Three blank filters were extracted for blank measurements. Duplicate OP measurements were performed for each PM sample. The DTT consumption rate (σ^DTT^, mM min^–1^) and the mass-normalized DTT activity (OP_m_^DTT^, pmol min^–1^ μg^–1^) of a sample were calculated using the following equations

1 $$\sigma^{\mathrm{DTT}}=-\sigma^{\mathrm{Abs}}\cdot \frac{C_{\mathrm{DTT},0}}{{\mathrm{Abs}}_{0}}$$

2 $${\mathrm{OP}}_{m}^{\mathrm{DTT}}=\frac{\sigma^{\mathrm{DTT},\mathrm{sample}}-\sigma^{\mathrm{DTT},\mathrm{blank}}}{C_{\mathrm{sample}}}$$

where σ^Abs^ (min^–1^) is the decay rate of TNB absorbance versus the incubation time, Abs_0_ is the initial absorbance of TNB extrapolated from the linear regression, *C*_DTT,0_ (mM) is the initial DTT molar concentration in the reaction cuvette, *C*_sample_ (g L^–1^) is the effective mass concentration of a PM sample. Measured values of OP_m_^DTT^ (pmol min^–1^ μg^–1^) and OP_v_^DTT^ (nmol min^–1^ m^–3^) are presented and discussed in this study, as they reflect the decay rates of DTT per PM mass and per air volume, respectively. The OP_m_^DTT^ is converted to OP_v_^DTT^ with the following equation

3 $${\mathrm{OP}}_{v}^{\mathrm{DTT}}={\mathrm{OP}}_{m}^{\mathrm{DTT}}\cdot \left[\mathrm{PM}\right]$$

where [PM] (μg m^–3^) is the mass concentration of bulk or size-segregated PM in the sampled air.

Additionally, for all the bulk PM_10_ samples, the 0.1 g L^–1^ PM_10_ water extracts were also treated with 4% (w/v) Chelex chelating ion-exchange resin (C7901, Sigma) by vortex shaking for 12 h at room temperature to remove transition metals. The differences in OP before and after the Chelex treatment were considered to be the OP contributed by the transition metals. In parallel with the Chelex treatment, 1 mmol L^–1^ ethylenediaminetetraacetic acid (EDTA) in a phosphate buffer was also mixed with the PM_10_ extracts for 10 min at room temperature as an alternative method to remove metals.^42,58^ The results of these two methods are compared and discussed in Section 3.4.

For all the bulk PM_10_ samples, cellular ROS was measured *in vitro* using 2′,7′-dichlorofluorescein diacetate (DCFH-DA), a fluorescent probe that reacts with a wide range of ROS. Procedures were consistent with our previous work.^39^ A549 cells were cultured in RPMI-1640 medium (Gibco, Thermo Fisher Scientific) supplemented with 5 μg mL^–1^ penicillin/streptomycin, 2 mM glutamine, and 10% fetal bovine serum (Biological Industries, Beit Ha-Emek, Israel) in a humidified incubator kept at 37 °C and with 5% CO_2_. Cells were dissociated from tissue culture flasks using 0.25% trypsin-EDTA (Gibco), resuspended in culture media, and seeded in 12-well plates at a density of 1.5 × 10^5^ cells/well 1 day before exposures. Buffered WS-PM_10_ extracts for cell exposures were prepared by extracting filters in a salts glucose medium (SGM) consisting of 500 mM HEPES, 1 M NaCl, 50 mM KCl, 20 mM CaCl_2_, and 50 mM dextrose at pH 7.2. Detailed procedures about the DCFH-DA assay are summarized in Text S1. The volume-based relative cellular ROS (ROS_v_, fold/(m^3^ air/mL H_2_O)) was calculated by the following equation to evaluate fold changes of fluorescence when PM_10_ in unit volume of air is extracted in unit volume of water

4 $${\mathrm{ROS}}_{v}=\frac{\left(F_{\mathrm{DCF}}-1\right)\cdot \left[{\mathrm{PM}}_{10}\right]}{100}+1$$

where *F*_DCF_ is the measured fold changes of fluorescence compared with the blank filter.

### 2.4 mRNA Expression

When PM-induced ROS cause oxidative stress in the human body, the nuclear factor erythroid 2-related factor 2 (Nrf2) pathway is a key antioxidant response to restore the redox homeostasis.^59^ Besides, specific PM components, such as polycyclic aromatic hydrocarbons (PAHs), can enhance xenobiotic activities in the body and result in metabolites that induce cellular oxidative stress and inflammation.^60,61^ These processes, referred to as phase I and II antioxidant systems, respectively, are associated with health effects of PM.^62^ To investigate the underlying mechanisms of the observed cellular ROS production, we used qPCR to measure changes in gene expression following exposure to the WS-PM_10_ extracts.^61^ Representative bulk PM_10_ samples collected in different periods were examined: 0503N (nonevent period), 0505D (mineral dust event), and 0508N1 (BB event). Detailed procedures about the gene expression measurements are summarized in Text S1. We measured genes in two categories: phase I/xenobiotic metabolism (cytochrome P450 enzymes, cyp1a1); and phase II detoxifying enzymes (Catalase, Cat; Glutamine cysteine ligase, Gclc; glutathione peroxidase, Gpx; heme oxygenase, HO-1; and superoxide dismutase 2, SOD2) which are regulated by the Nrf2. The volume-based relative gene expression (Gene_v_, fold/(m^3^ air/mL H_2_O)) was calculated with the following equation

5 $${\mathrm{Gene}}_{v}=\frac{\left(F_{\mathrm{PCR}}-1\right)\cdot \left[{\mathrm{PM}}_{10}\right]}{100}+1$$

where *F*_PCR_ is the measured fold changes of gene expression compared with the blank filter. Both cellular ROS and mRNA assays were conducted in at least two independent experiments, and each experiment was conducted in triplicate.

### 2.5 Source Apportionment of PM_10_ Samples

The PM_10_ source apportionment was conducted using the PMF receptor model and the multilinear engine (ME-2) algorithm.^63^ The analysis was conducted with EPA-PMF 5.0 software. In this study, the input data consisted of a 25 × 25 matrix, with 25 bulk PM_10_ samples as rows and 25 chemical species as columns. In addition to the measured concentrations of PM_10_, WSOC, WSOM, *f*_44_, *f*_60_, three inorganic anions, and 16 elements as aforementioned, the oxidant O_*x*_ (O_3_ + NO_2_) was also included to enhance the recognition of PM_10_ from secondary formation processes. Details on specific settings, constraints, and diagnoses are summarized in Text S2.

The input matrix in this study has a relatively small size of 25 × 25. Although PMF has been shown to produce reasonable results for datasets of comparable sizes,^19,64^ caution is required when interpreting the results.^65^ For our dataset, constrained bootstrap factors and their corresponding base factors have mapping rates larger than 88%, indicating the stability of the PMF solution. Principal Component Analysis (PCA) was also employed to identify significant sources of PM_10_ (Text S2), and it resolved six factors that closely matched the PMF factors (Table S3 vs Table S4). PMF includes a non-negativity constraint and weighs the data using uncertainty values, which are advantages against PCA.^66^ Based on these strengths, the following discussion focuses solely on the PMF results.

The apportionments of OP^DTT^ and cellular ROS to different pollution sources were conducted with a widely used multilinear regression (MLR) method.^7,67,68^ The details of this method are summarized in Text S4.

### 2.6 Dosimetry of Particle OP^DTT^

The OP^DTT^ dose (OP_dose,T_, in nmol min^–1^ h^–1^) in the human respiratory tract exposed to the WS-PM_10_ for a unit of time was calculated using the following equation

6 $${\mathrm{OP}}_{\mathrm{dose},T}=\sum {\mathrm{OP}}_{V,i}^{\mathrm{DTT}}\cdot {\mathrm{DF}}_{\mathrm{ij}}\cdot \mathrm{TV}\cdot \mathrm{RF}$$

where DF, TV, and RF are the deposition fraction, tidal volume (m^3^), and respiratory frequency (h^–1^), respectively; *i* is the PM size bin separated by the cascade impactor as demonstrated in Section 2.1; *j* is the specific region in the human respiratory tract, including the pulmonary region, tracheobronchial region, and head airways. The DF_*i,j*_ of PM in a healthy adult at rest was predicted using the Multiple-path Particle Dosimetry Model (MPPD, version 3.04).^69^ Parameters used for the OP_dose,T_ calculation are summarized in Text S5.

To evaluate the intrinsic properties of different pollution sources, the PM_10_ mass-based OP^DTT^ dose (OP_dose,M_, in pmol min^–1^ μg^–1^) was calculated as

7 $${\mathrm{OP}}_{\mathrm{dose},M}=\frac{{\mathrm{OP}}_{\mathrm{dose},T}}{\mathrm{TV}\cdot \mathrm{RF}\cdot \left[{\mathrm{PM}}_{10}\right]}$$

where [PM_10_] is mass concentration of PM_10_ in the sampled air.

## 3 Results and Discussion

### 3.1 Linking Sources and Single Species to OP^DTT^ and ROS

Based on chemical composition data, six distinct sources were identified in our dataset with the PMF analysis. The PMF factors were interpreted as different pollution sources according to their characteristic marker species and temporal patterns, which are summarized in Text S3. As a result, these sources were recognized: Mineral dust, biomass burning, industrial dust, traffic, secondary formation, and marine.

With a MLR method (Text S4), mineral dust, industrial dust, BB, and the traffic factors were correlated with the OP_v_^DTT^, while BB and traffic factors were correlated with the ROS_v_. As Figure 1 shows, the toxicity of WS-PM_10_ measured by these two endpoints were linked with pollution sources, and the results are consistent with observed events. Specifically, during the dust storm event on daytime of May 5, mineral dust explained 83% of OP_v_^DTT^, while its contribution to ROS_v_ could not be quantified due to its insignificant correlation with ROS_v_ (*p* < 0.05). In the evening of the bonfire festival on May 8, BB contributed 80 and 88% to OP_v_^DTT^ and ROS_v_, respectively. According to the source apportionment, the role of anthropogenic activities in nonevent periods was important: BB, traffic, and industrial dust explained 65% of OP_v_^DTT^, while traffic and BB explained 79% of ROS_v_.

**Figure 1:**
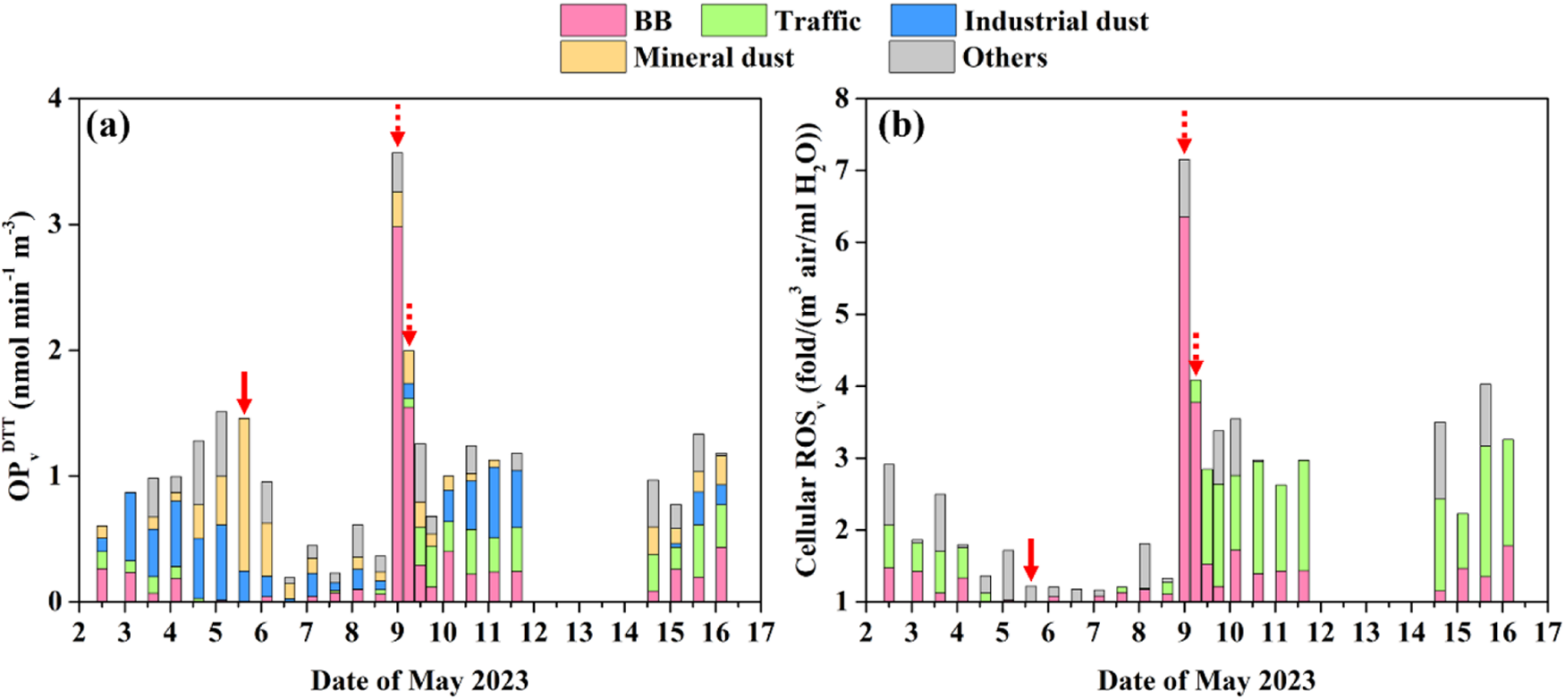
Allocation of WS-PM_10_ induced (a) OP_v_^DTT^ and (b) cellular ROS_v_ to different pollution sources. The solid and dashed arrows denote the mineral dust event and the BB event, respectively. The unit of ROS_v_ mean fold changes of fluorescence when PM_10_ in unit volume of air is extracted in unit volume of water. The cellular ROS_v_ was measured independently two times with three replicas of each treatment. Raw fluorescence data were normalized to the corresponding blank fluorescence signal from the same experiment. A dashed line is plotted as a reference at *y* = 1. The standard deviation is shown as shadow. The solid and dashed arrows denote the mineral dust event and BB event, respectively.

The Pearson’s correlation coefficients of chemical species with OP_v_^DTT^ and ROS_v_ are summarized in Table 1. Marker species for mineral dust and marine sources were not significantly correlated with either OP_v_^DTT^ or ROS_v_, including [PM_10_], a basic measurement and mineral dust marker. This indicates that [PM_10_] alone does not trace the health risks of particles, consistent with studies in other mineral dust-affected areas.^17,18^ The secondary formation marker *f*_44_ negatively correlated with OP_v_^DTT^ and ROS_v_, possibly because the primary BB particles with relatively high toxicity had relatively low values of *f*_44_.

**Table 1:** Pearson’s Correlation Coefficients between PM_10_ Chemical Data and Acellular/Cellular OP Parameters^a^

		OP_v_^DTT^		ROS_v_	
marked source	species	Pearson’s *r*	significance^b^	Pearson’s *r*	significance
mineral dust	PM_10_				
	Al				
	Ca				
marine	Na				
	Mg				
	chloride				
industrial dust	V				
	Ba	0.47	*		
BB	*f*_60_	0.78	***	0.66	***
	K	0.84	***	0.60	***
	WSOC	0.89	***	0.66	***
	Zn	0.83	***	0.81	***
	As	0.85	***	0.75	***
	Cd	0.84	***	0.78	***
traffic	Fe			0.41	*
	Co			0.57	***
	Ni				
	Cu			0.40	***
	Pb	0.52	**	0.53	**
secondary formation	*f*_44_	–0.61	***	–0.47	*
	O_*x*_				
nonspecific^c^	Mn	0.79	***	0.82	***
	nitrate	0.49	*		
	sulfate			0.45	*

a Blank means no significant correlation (*p* > 0.05) was observed.

b ***: *p* < 0.05; *p* < 0.01, *: *p* < 0.05.

c Single factor can explain less than 30% contribution for these species.

It is also noted that several HM species, which are marker species for anthropogenic pollution sources, were positively correlated with OP_v_^DTT^ and ROS_v_. This is consistent with the PMF results, which also emphasize the role of anthropogenic sources. Specifically, Zn, Cd and As reached peak values during the anthropogenic BB event (Text S3 and Figure S1), and they positively correlated with both OP_v_^DTT^ and ROS_v_. Ba, Fe, Co, Cu, and Pb were marker species for industrial or traffic sources (Text S3 and Table S3), and they correlated with either OP_v_^DTT^ or ROS_v_, or both of them. This suggest that HMs could be one of important chemical components linking anthropogenic activities with WS-PM_10_ toxicity in this Eastern Mediterranean site.

### 3.2 The Role of HMs in OP_v_^DTT^

We assessed the contribution of HMs to OP_v_^DTT^ for each filter sample by removing them in the water extracts and then measuring the difference in OP_v_^DTT^. Two methods were used in parallel to remove them, namely the Chelex ion exchange and the EDTA chelation methods. As shown in Figure 2, the EDTA chelation method reported higher OP_v_^DTT^ values for HMs than the Chelex ion exchange method in 22 out of the 25 samples. During the BB event, the EDTA chelation method indicated that 94–96% of OP_v_^DTT^ were explained by HMs. However, WSOC positively correlated with OP_v_^DTT^ (Table 1), and its content in PM_10_ during the BB event was 1.1 times higher than the average value in other periods. Therefore, the performance of the EDTA chelation method was questioned.

**Figure 2:**
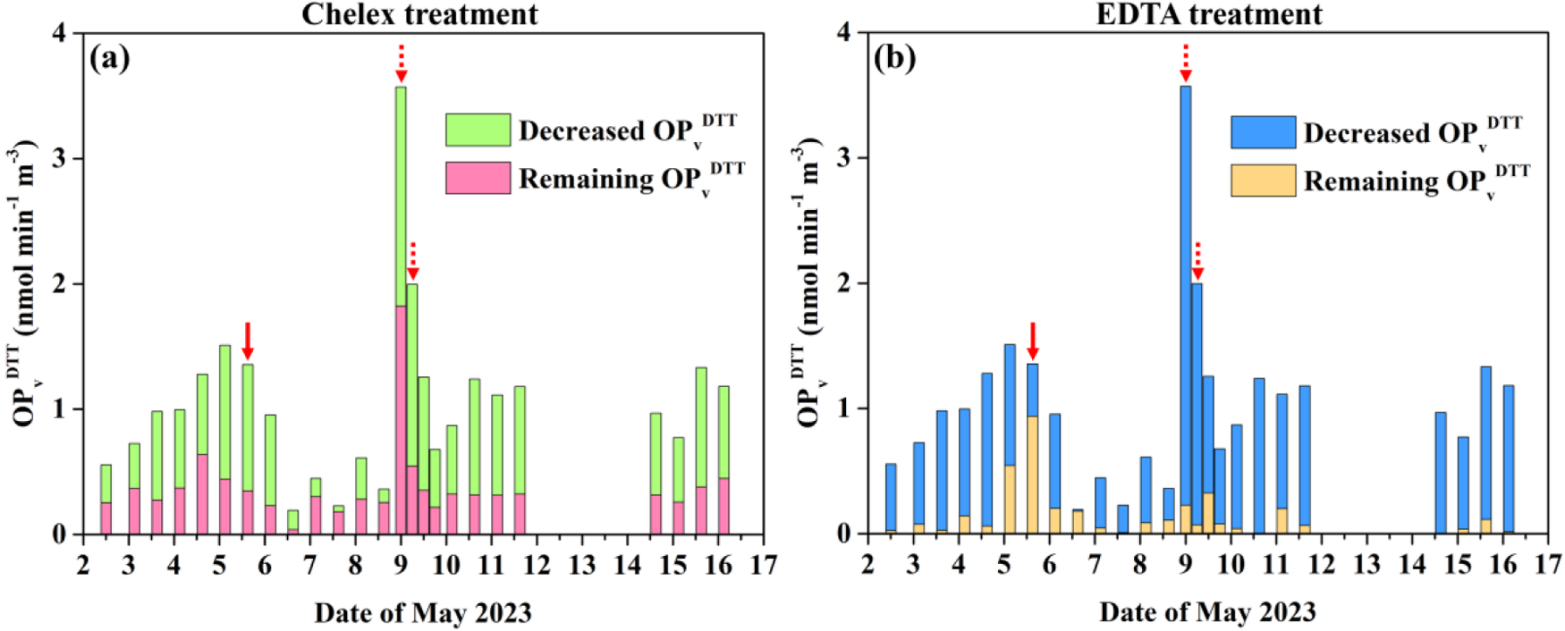
Decreased and remaining OP_v_^DTT^ with the method of (a) Chelex ion exchange, (b) EDTA chelation. The solid and dashed arrows denote the mineral dust event and the BB event, respectively.

To determine the interaction of EDTA with possible WS components in particles, the OP activities of several mixed standard solutions were measured (Table 2). Two representative organic species were chosen: the 2-methoxy-1,4-hydroquinone (MHQ) is an phenolic species in the BB particles;^39,70^ and 1,4-naphthaquinone (1,4-NQ) is a PAH oxidation product in ambient particles.^71,72^ Fe^3+^ and Cu^2+^ are representative HM species that can produce ROS in aqueous solution through Fenton-like reactions.^36,73^ Positive DTT consumption rates were observed in mixtures with metal/organic mass ratio of 1:50 (Group B, D, J, and L). After the EDTA treatment was added to those combinations of metal/organic mixture (Group C, E, K, and M), the DTT consumptions decreased below the detection limit. These observations are in line with the overestimation of HMs’ OP^DTT^ in most PM_10_ samples using the EDTA treatment. The EDTA can enhance the efficiency of Fenton reaction, and the formed hydroxy radical can then consume the organics in the solution,^74,75^ which might explain the decreased OP^DTT^.

**Table 2:** DTT Consumption Rates of Mixed Standard Solutions^a^

		added chemicals (mg L^–1^)			
solution label	base solution^b^	Fe^3+^	Cu^2+^	EDTA	DTT consumption rate (μM min^–1^)
A	1 mg L^–1^ MHQ	0	0	0	0.15 ± 0.01
B	1 mg L^–1^ MHQ	0.02	0	0	0.33 ± 0.01
C	1 mg L^–1^ MHQ	0.02	0	234	LDL^c^
D	1 mg L^–1^ MHQ	0	0.02	0	1.03 ± 0.07
E	1 mg L^–1^ MHQ	0	0.02	234	LDL
F	1 mg L^–1^ MHQ	1	0	0	1.39 ± 0.12
G	1 mg L^–1^ MHQ	1	0	234	2.70 ± 0.05
H	1 mg L^–1^ MHQ	0	0	234	LDL
I	0.15 mg L^–1^ 1,4-NQ	0	0	0	0.52 ± 0.03
J	0.15 mg L^–1^ 1,4-NQ	0.02	0	0	0.67 ± 0.03
K	0.15 mg L^–1^ 1,4-NQ	0.02	0	234	LDL
L	0.15 mg L^–1^ 1,4-NQ	0	0.02	0	0.78 ± 0.03
M	0.15 mg L^–1^ 1,4-NQ	0	0.02	234	LDL
N	0.15 mg L^–1^ 1,4-NQ	0	0	234	LDL

a The concentrations in the incubation solution are shown.

b MHQ: 2-methoxy-1.4-hydroquinone; 1,4-NQ: 1,4-naphthaquinone. The chosen concentrations of organics ensured that less than 25% of the initial DTT was consumed after 40 min of reaction.

c LDL: lower than the detection limit.

It is also observed that the OP_v_^DTT^ of HMs in the mineral dust event samples measured by the EDTA method (0.42 ± 0.03 nmol min^–1^ m^–3^) was less than the results from the Chelex method (1.01 ± 0.20 nmol min^–1^ m^–3^). To mimic the high metal content in the desert dust, we set the Groups F and G in which the mass ratio of metal to MHQ were 1:1. The DTT consumption was accelerated in Group G compared with Group F, in line with the results of the mineral dust sample. EDTA also affected DTT consumption rates of organic pollutants in the absence of metals: it decreased the OP of both MHQ and 1,4-NQ without metals (Group H and N). It is noted that EDTA can reduce the OP of several other quinone species,^76^ but the mechanism is not known yet. Overall, the EDTA is unsuitable for quantifying the OP of metal species in mixtures containing organics since it has complex interactions with OP-related species. In contrast, the Chelex ion exchange method was found to have no influence on the OP of either MHQ or 1,4-NQ (Table S8). Therefore, only the results measured by the Chelex method are discussed hereinafter, although whether Chelex can interact with other organic species has not been investigated.

As shown in Figure 2a, HMs contributed 61 ± 16% of OP_v_^DTT^ throughout the campaign. The large contribution of HMs to OP_v_^DTT^ has been observed in other urban sampling sites.^77,78^ The average contribution in anthropogenic-dominant samples (61 ± 16%) and in the remaining samples (61 ± 18%) were almost similar, indicating HMs’ important role in acellular OP under different pollution scenarios. During the mineral dust event, the contribution of metals was at a relatively high level (74%). Therefore, HMs may also be important chemical components linking desert dust with OP_v_^DTT^.

The OP^DTT^ of single or mixed HM species in water solution has been previously explored.^51,52,76,79^ With the mass concentration measured with ICP-MS, the reconstructed OP_v_^DTT^ of metals (OP_metals_^R^) was calculated as described in the Text S6. We compared the measured (OP_metals_^M^, Chelex ion exchange method) and the reconstructed OP_v_^DTT^ of HMs in Figure 3. It turns out that the OP_metals_^R^ traced the general trend of OP_metals_^M^ well during the entire period, but underestimated the absolute value of OP_metals_^M^ during the BB event. Figure 3(b) shows that the difference between OP_metals_^M^ and OP_metals_^R^ was related to the proportion of HMs in OP_v_^DTT^. When the OP_metals_^R^ consisted of more than 60% of OP_v_^DTT^, the difference between OP_metals_^M^ and OP_metals_^R^ remained at a reasonably low level. When the proportion of OP_metals_^R^ in OP_v_^DTT^ was less than 60%, the difference between OP_metals_^M^ and OP_metals_^R^ increased as HMs contributed less. This is because the OP_metals_^R^ does not consider the interaction between HMs and nonmetals, while the OP_metals_^M^ does. Therefore, a synergistic effect between HMs and nonmetals in inducing OP^DTT^ is shown for the ambient PM_10_ in this region, especially at the BB event. This is consistent with previous findings that the humic-like substances and specific quinone species in BB particles add to the OP^DTT^ activity of several HM species.^79–81^

**Figure 3:**
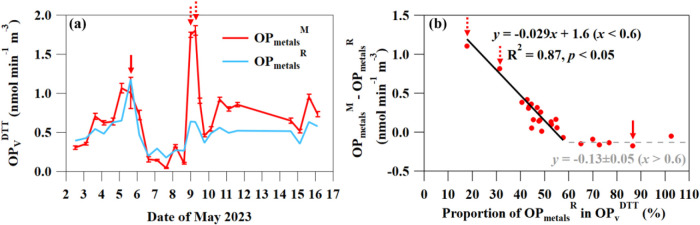
(a) Time series of OP_metals_^M^ and OP_metals_^R^; (b) the relationship between (OP_metals_^M^–OP_metals_^R^) and the proportion of OP_metals_^R^ in OP_v_^DTT^. The OP_metals_^M^ was quantified by the Chelex ion exchange method. The solid and dashed arrows denote the mineral dust event and the BB event, respectively.

### 3.3 Aerosol Dosimetry Analysis during Specific Sampling Periods

Seven size-segregated samples were collected during the campaign, and they were classified into four sampling periods according to the source apportionment results based on their simultaneous bulk PM_10_ samples (Figure 1 and Table S1): Regular period, mineral dust event, BB event, and industry-affected period. Size-dependence of OP^DTT^ and aerosol dosimetry analysis were investigated to characterize the acellular OP of WS-PM_10_ in different periods.

The size-dependent OP_m_^DTT^ and OP_v_^DTT^ for WS-PM in each period are shown in Figure S7. For each period, the total OP_dose,T_ calculated based on the size-resolved OP_v_^DTT^ and their corresponding deposition rates in the human respiratory tract are summarized in Figure 4a. The total OP_dose,T_ for the regular period was approximately 0.2 nmol min^–1^ h^–1^, close to the background OP_dose,T_ measured in another Mediterranean region.^31^ The total OP_dose,T_ during the BB event and the mineral dust event were 2.2 and 0.8 times larger than during the regular period, respectively. The industry-affected particles (0.22 nmol min^–1^ h^–1^) had a comparable total OP_dose,T_ to the regular period particles (0.21 nmol min^–1^ h^–1^). Detailed size-resolved OP_dose,T_ in different regions are shown in Figures S8–S11. For the regular period particles and BB event particles, the <0.49 μm size bin was the dominant contributor for OP_dose,T_ deposited in the pulmonary and tracheobronchial regions, and the deposition fractions in the thoracic region ranged within 35.8–41.5%. The dust event particles and industry-affected particles were both characterized by large contributions from the 3.0–7.2 μm to the head airway OP_dose,T_, and the deposition fractions in the thoracic region decreased to 28.6–33.7%. More severe health-related outcome is expected if particles penetrate deeper into the human respiratory tract.^82^ When considering the deposited OP_dose,T_ in the pulmonary region, the BB event, the mineral dust event, and the industrial influence increased it by 209%, 47%, and 4%, respectively, compared with regular period particles.

**Figure 4:**
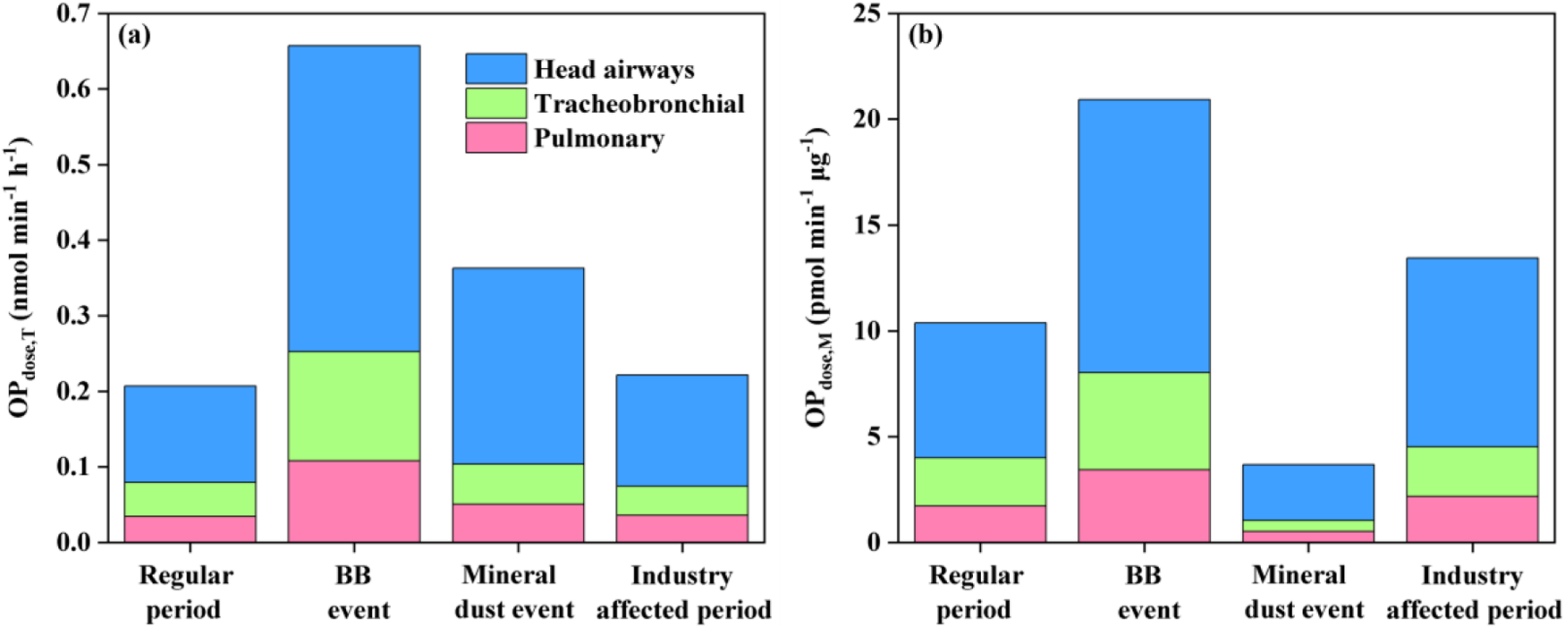
OP^DTT^ dosage in the human respiratory tract in different periods per unit (a) time, and (b) mass of PM_10_.

Figure 4b shows the calculated exposed OP^DTT^ dosage in the human respiratory tract per unit mass of PM_10_ in the ambient air, which is an intrinsic toxicological property of PM_10_ regardless of mass concentrations in different scenarios. The PM_10_ in the BB event, mineral dust event, and industry-affected period had total OP_dose,M_ that were 202, 35, 135% of the OP exposure in the regular period, respectively. The potential health risks of the WS-PM_10_ emitted from anthropogenic BB activities were highlighted with an acellular DTT assay.

### 3.4 Comparison of WS-PM_10_ Toxicity Measured with OP^DTT^, Cellular ROS, and Gene Expression

The OP_v_^DTT^ and ROS_v_ results for WS-PM_10_ were correlated with each other (Pearson’s *r* = 0.75, *p* < 0.05) throughout the entire sampling period, supporting using the acellular DTT assay as a preliminary health risk assessment. Specifically, anthropogenic sources (BB, traffic, and industrial dust) accounted for >50% of both OP_v_^DTT^ and ROS_v_ in 14 samples (anthropogenic-dominant), and the correlation between OP_v_^DTT^ and ROS_v_ was particularly significant (Figure 5, Pearson’s *r* = 0.87, *p* < 0.05); while in the remaining 11 samples, the correlation was insignificant (Figure 5, Pearson’s *r* = 0.25, *p* > 0.05). Consistency and divergence between acellular assays and cellular assays have been reported in ambient PM from other areas.^40,41^ In this study, samples from the BB event and the mineral dust event exemplify these two opposite cases.

**Figure 5:**
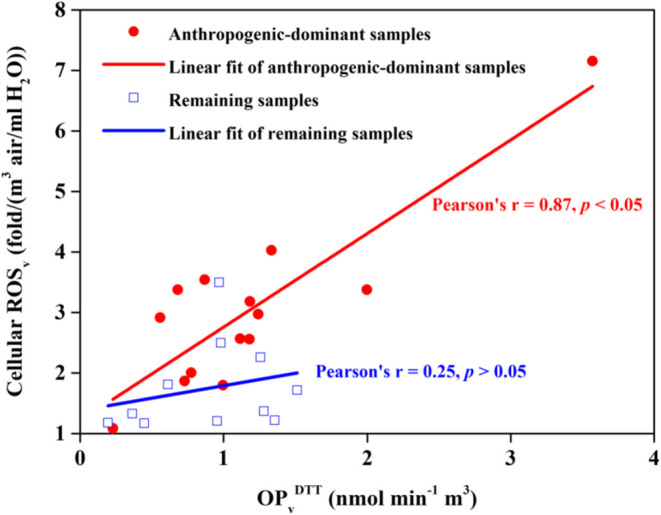
Correlation between OP_v_^DTT^ and cellular ROS_v_ in anthropogenic-dominant samples and the remaining samples. Anthropogenic-dominant: anthropogenic sources (BB, traffic, and industrial dust) accounted for >50% of both OP_v_^DTT^ and ROS_v_ in 14 samples, and they are listed in Table S1.

Both OP_v_^DTT^ and ROS_v_ reached peak values during the BB event on the night of May 8, suggesting the potential acute health effects of anthropogenic BB activities. During the BB event, both PM_10_ concentration (68.7 ± 6.4 μg m^–3^) and OP_m_^DTT^ (39.9 ± 12.4 pmol min^–1^ μg^–1^) were significantly elevated compared to the nonevent period. This resulted in an OP_v_^DTT^ value of 2.78 ± 1.11 nmol min^–1^ m^–3^, which is 3.2 times that of the nonevent period. The increased OP^DTT^ of ambient particles caused by BB has been extensively documented in recent years.^41,83,84^ The OP_m_^DTT^ and OP_v_^DTT^ values of the BB event in this study fell within the range of reported literature values (Table S5).

The mineral dust event exhibited lower OP_m_^DTT^ (6.2 ± 0.4 pmol min^–1^ μg^–1^) than the nonevent period (20.9 ± 7.2 pmol min^–1^ μg^–1^), which is consistent with studies conducted in other desert dust-influenced Middle East and Mediterranean regions.^16–18^ Because of the surge of PM_10_ mass concentration at the mineral dust event, the OP_v_^DTT^ increased to 1.36 ± 0.09 nmol min^–1^ m^–3^, which was higher than the nonevent periods (0.88 ± 0.37 nmol min^–1^ m^–3^). In contrast, the cellular ROS_v_ during the mineral dust event remained relatively low (1.22 fold/(m^3^ air/ml H_2_O), Figure 1b) compared with the nonevent period (2.27 ± 0.90 fold/(m^3^ air/ml H_2_O)). This suggests that OP_v_^DTT^ may not be directly used to predict the trend of cellular ROS_v_ measured by the DCFH-DA assay for PM_10_ affected by mineral dust.

In addition to OP^DTT^ and cellular ROS measurements, we exposed the WS extracts to A549 cells to observe gene expression, which can indicate toxicity pathways. The aryl hydrocarbon receptor (AhR) enzyme involved in xenobiotic metabolism, Cyp1a1, can be activated by PAHs to form metabolites such as quinones that contribute to ROS formation.^60,61,85,86^ As shown in Figure 6, the WS components of representative PM_10_ upregulated the expression of Cyp1a1 in the following order: BB event > nonevent > mineral dust event, which is consistent with the trend of cellular ROS_v_. Burning processes can emit significant amounts of PAHs.^87^ According to the PMF analysis, the representative nonperiod event sample (0503N) belongs to the anthropogenic-dominant samples, while the mineral dust sample (0505D) does not. Therefore, the source contribution likely accounts for the pronounced differences in Cyp1a1 expression observed in these periods. It is also noted that unoxidized PAHs typically have minimal DTT activity,^36,88^ which may explain the difference between the OP_v_^DTT^ and cellular ROS_v_ in samples that were less influenced by anthropogenic sources. We also investigated genes that can protect cells from oxidative stress in different ways: HO1 catalyzes the degradation of heme and forms CO as an antioxidant;^89^ Cat catalyzes the decomposition of H_2_O_2_ to water and oxygen; and Gclc catalyzes the rate-limiting step in the production of glutathione, a cellular antioxidant.^90^ Notably, the BB event PM_10_ significantly upregulated all these genes’ expressions compared to the blank, while the mineral dust event PM_10_ did not induce a significant response for any of them (Figure 6b–d). Gpx and SOD2 genes were not significantly upregulated at the explored dosage in any event period. Overall, PM_10_ from the BB event induced the greatest Cyp1a1 gene expression per unit volume of inhaled air, significantly elevated cellular ROS_v_ formation, and strongly stimulated the secretion of detoxifying enzymes. In contrast, the mineral dust event sample generated minimal cellular ROS_v_ and did not elicit the investigated defense mechanisms.

**Figure 6:**
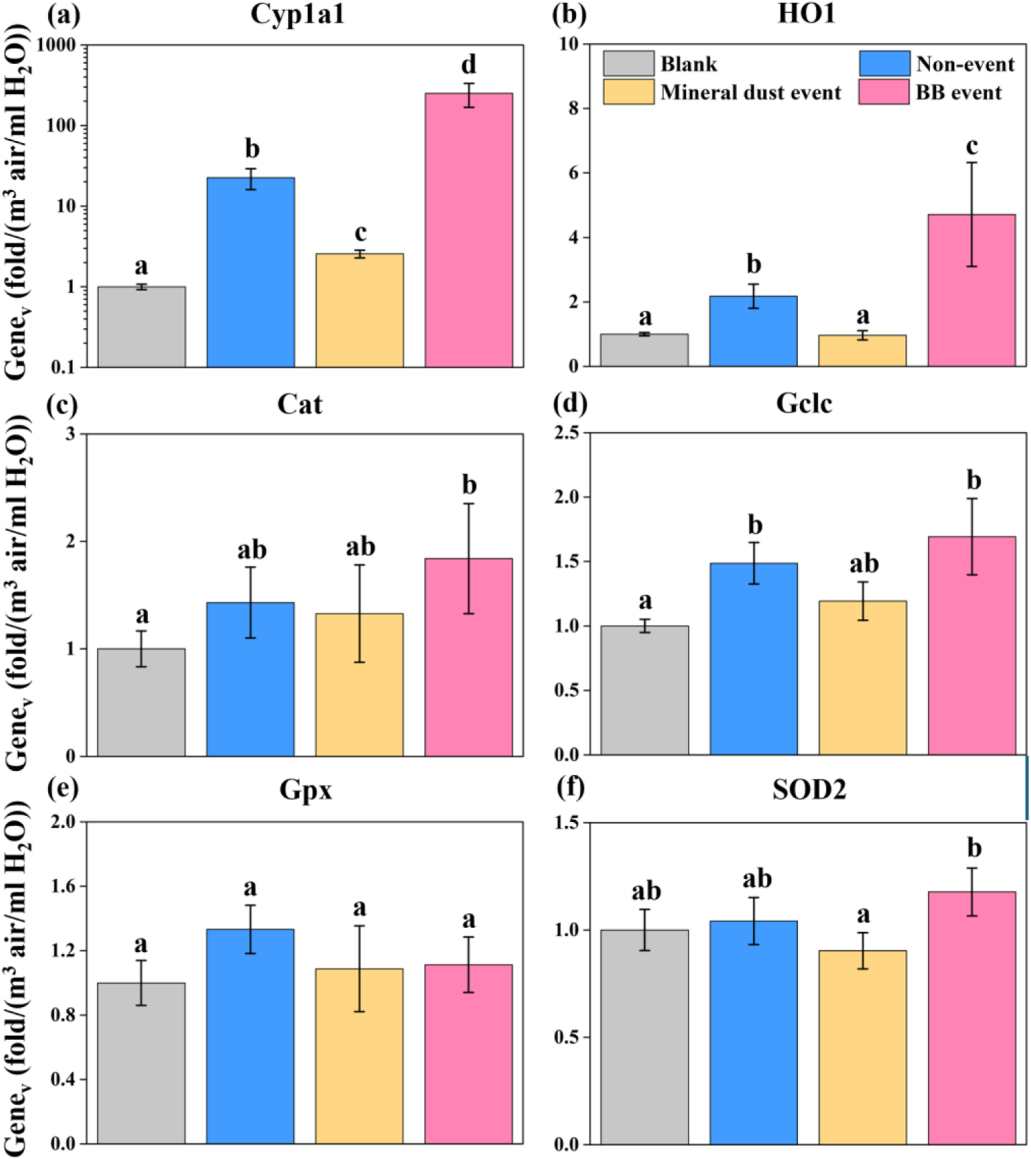
Gene_v_ following exposure to WS-PM_10_ for (a) Cyp1a1, (b) Cat, (c) Gclc, (d) Gpx, (e) HO1, and (f) SOD2 relative to the control group. The unit of gene_v_ means fold changes of gene expression when PM_10_ in unit volume of air is extracted in unit volume of water. Experiments were performed independently two times with three replicas of each treatment. Error bars indicate standard error. Bars with different letters have significantly (*p* < 0.05) different mean values, which were assessed with one-way ANOVA followed by post hoc tests with Tukey HSD (under variance homogeneity) or with Tamhane’s T2 (under variance heterogeneity).

## 4 Conclusions

In a two-week campaign conducted in the Eastern Mediterranean Region at the onset of the dry season, the OP_v_^DTT^ and ROS_v_ of WS-PM_10_ were attributed to different pollution sources using the PMF-MLR method and were correlated with multiple water-soluble chemical components. Both analyses highlighted the significant influence of anthropogenic activities on the health risks of WS-PM_10_, although natural mineral dust contributed to ∼50% of the PM_10_ burden during the campaign (Figure S6).

HMs were major contributors to OP_v_^DTT^ during the campaign, linking anthropogenic sources and desert dust to acellular OP. A synergistic effect between HMs and nonmetals in inducing acellular OP is shown when HMs explain <60% of OP_v_^DTT^. Methodologically, the Chelex ion exchange method reliably quantified the contribution of HMs to OP_v_^DTT^, while EDTA treatment introduced artifacts, leading to biases in OP^DTT^ interpretation. Specifically, the EDTA treatment overestimates and underestimates the metals’ OP_v_^DTT^ during the BB event and the mineral dust event, respectively. Given these inconsistencies, we recommend the Chelex method over EDTA for metal attribution in the DTT assay, and caution is advised when applying EDTA in other steps of the DTT assay as well.

In 14 out of 25 anthropogenic-dominant samples, the OP_v_^DTT^ and ROS_v_ results were positively correlated (*p* < 0.05), supporting the acellular DTT assay as a preliminary health risk assessment when the air quality is significantly influenced by anthropogenic activities. A bonfire festival was an example of such an anthropogenic-dominant period. OP_v_^DTT^ and ROS_v_ both reached peak values during this period, the gene expression showed significant upregulation of several oxidative stress-related genes (Cyp1a1, HO1, Cat, Gclc). Aerosol dosimetry analysis found that the pulmonary OP_dose,T_ during the BB event was approximately two times higher than the regular period. The increased health risks from WS-PM_10_ call for greater public awareness of government instructions during the bonfire festival.

In the remaining 11 samples where the influences of anthropogenic sources were less significant, OP_v_^DTT^ and ROS_v_ results were not significantly correlated (*p* > 0.05). A natural mineral dust event exemplifies one such period, when OP_v_^DTT^ was higher than during nonevent periods, while the opposite was true for ROS_v_. Specifically, the pulmonary OP_dose,T_ during the dust event period was 47% higher than the regular period. Measurements on gene expression found the downregulation of Cyp1a1 during the dust event compared with during the nonevent periods. Cyp1a1 can be triggered by aromatic compounds (e.g., PAHs) to generate cellular ROS. Therefore, the relatively low organic content of the mineral dust event sample could be one of reasons for its relatively low cellular ROS_v_.

Given the study’s small dataset, the use of a single exposure dose in cellular assays, and its focus on WS-PM_10_, further work is needed to improve health risk assessments in the Eastern Mediterranean and to better understand the contributions of different regional pollution sources. This will include long-term monitoring, dose-dependent studies, and investigations of insoluble PM fractions.

## Supplementary Material



## Notes

Experimental and modeling details on filter sampling, acellular/cellular assays, PMF/PCA analysis, aerosol dosimetry analysis for OP^DTT^, and the reconstruction of metals’ OP^DTT^ (PDF)
