# Supplementary material for: Investigating the Oxidative Potential and In Vitro Toxicity of Ambient Water-Soluble PM10 in an Eastern Mediterranean Site
Source: ACS EST Air. 2025 Jun 16;2(7):1326–38. doi: 10.1021/acsestair.5c00085 (PMC12261277; doi:10.1021/acsestair.5c00085)
Supplement: Supplementary file 1 [file ea5c00085_si_001.pdf]

Supplementary information of

Investigating the oxidative potential and *in vitro* toxicity of ambient water-soluble PM_{10} in an Eastern Mediterranean site

Zheng Fang [a], Alexandra Lai [a], Eli Windwer [a], Michal Pardo [a], Chunlin Li [b], Ajith Thenoor Chandran [a], Alexander Laskin [c], Yinon Rudich [a,*]

[a] Department of Earth and Planetary Sciences, Weizmann Institute of Science, Rehovot 76100, Israel

[b] College of Environmental Science and Engineering, Tongji University, Shanghai 200072, China

[c] Department of Chemistry, Purdue University, West Lafayette, Indiana 47907, United States

***Correspondence to:** Yinon Rudich (yinon.rudich@weizmann.ac.il)

This supporting information file contains texts, table, and figures as follows:

- Text S1: DCFH-DA assay and cell death assay.
- Text S2: PMF setup and comparison with PCA.
- Text S3: Interpretation of PMF source apportionment results.
- Text S4: Linking OP_v^{DTT} and ROS_v of WS-PM_{10} with pollution sources.
- Text S5: Parameters used for $OP_{dose,T}$ estimation.
- Text S6: Method to reconstruct OP_v^{DTT} of metals.
- Table S1: Basic information of PM_{10} filter samples.
- Table S2: Cell death rate of A549 after exposure to 0.1 g L^{-1} PM_{10} extract for 24 h.
- Table S3: Summary of the characteristic marker species used to identify PMF factors.
- Table S4: Loadings of chemical species in the factors resolved by the PCA.
- Table S5: PM concentration and OP^{DTT} of WS components in different sampling periods.
- Table S6: Regional deposition efficiency in the human respiratory tract as predicted from the MPPD model for particle in different size ranges.
- Table S7: Regression equations for the DTT decay rate of individual metal species.
- Table S8: The influence of Chelex treatment to 2-methoxy-1.4-hydroquinone or 1,4-naphthaquinone.
- Fig. S1: The time series of abundant species in the BB factor from the PMF analysis.
- Fig. S2: The profile of factors derived from the PMF analysis.
- Fig. S3: The time series of factor contribution derived from the PMF analysis.
- Fig. S4: The IM and IS when different factor numbers are used in the PMF model.
- Fig. S5: The wind rose for each filter sampling period.

- Fig. S6: (a) The mass ratio of PMF factors for each filter sampling period; (b) The contribution of PMF factors to PM_{10} during the entire sampling period.
- Fig. S7 OP_m^{DTT} and OP_v^{DTT} for size-resolved fractions of WS-PM.
- Fig. S8: Size-dependent $OP_{dose,T}$ in the entire human respiratory tract.
- Fig. S9: Size-dependent $OP_{dose,T}$ in the human pulmonary region.
- Fig. S10: Size-dependent $OP_{dose,T}$ in the human tracheobronchial region.
- Fig. S11: Size-dependent $OP_{dose,T}$ in the human head airways.

Text S1: DCFH-DA assay, cell death assay, and PCR

A549 cells were incubated with 10 μM DCFH-DA in the dark at 37 °C for 30 minutes, rinsed twice with PBS, and then incubated with 0.1 g L^{-1} PM_{10} filter extracts in SGM for 24 hours. Fluorescence was measured by flow cytometry with excitation and emission wavelengths of 488 and 529 nm, respectively. A blank filter sample was extracted and measured for cellular ROS in the same way, and all measured cellular ROS levels of PM_{10} samples were normalized to the blank value. Cellular death after exposure to 0.1 g L^{-1} PM_{10} filter extracts for 24 h was measured by propidium iodide (PI). As shown in Table S2, no tested PM_{10} samples showed significantly different cell death rates compared to the blank filter sample, so the measured cellular ROS in the investigated samples were not affected by cell death.

PI is a membrane-impermeable DNA-intercalating dye that is excluded by viable cells. Following a 24-hour exposure to PM extracts, cells were dissociated with trypsin, resuspended in phosphate-buffered saline (PBS) with PI, and incubated at room temperature in the dark for 15 minutes. Fluorescence was measured by flow cytometry (Amnis CellStream, Luminex, USA) with excitation and emission wavelengths of 488 and 610 nm, respectively.

For the gene expression measurement, A549 cells were exposed to 0.1 g L^{-1} PM_{10} extracts for 5 hours and then treated with a RNeasy Mini Kit (QIAGEN, Hilden, Germany) and RNase-free DNase for mRNA isolation. RNA was quantified using a NanoDrop spectrophotometer (Thermo Fisher) and 500 ng of mRNA was converted to cDNA using random hexamers (Invitrogen). The mRNA expression was quantified by real-time PCR using a Fast SYBR Green PCR mix (Applied Biosystems) with a StepOnePlus PCR

instrument (Applied Biosystems). β-Actin was used as a reference gene, and as in the cellular ROS analysis, all data were normalized to the recorded values obtained using a blank filter that was extracted and analyzed alongside with the PM_{10} samples.

Text S2: PMF setup and comparison with PCA

As part of input for the PMF analysis, the uncertainty σ_{ij} (where *i* represents the sample, and *j* represents the species) was calculated by the following equation:[1,2]

$$\sigma_{ij}=\sqrt{\mathrm{DL}_j^2+(\mathrm{CV}_{ij}\times x_{ij})^2+(a\times x_{ij})^2} \qquad \text{Eq.S1}$$

where DL_j represents the detection limit that is calculated as twice the standard deviation of the blank sample; CV_{ij} is the coefficient of variation for repeated measurements; *a* is the additional uncertainty propagated by filter sampling (assumed 5%), filter extraction (assumed 5%), and analytical uncertainty for each technique. Specifically, for nickel, since its signal-to-noise ratio was less than 2, it was classified as a "weak" variable, and additional uncertainty was applied to it by the EPA-PMF software.

The PMF model was run with the factor number ranging from 3 to 10. The maximum individual column mean (IM) and the maximum individual column standard deviation (IS) indicate the average residual and the standard deviation of the residual for the least-fitted species, respectively, and they were used to determine the minimum number of factors that should be used.[3] As shown in Fig. S4, both IM and IS decrease when the factor number increases from 3 to 6, and then reach a plateau when the number of factors is more than 6. Lower IM and IS indicate smaller residuals and higher precision of the least-fitted species, respectively. Therefore, at least 6 factors are recommended to be included in the PMF analysis. For our dataset, 7 or more factors caused redundant or unexplained factors (mapping rate below 80%) diagnosed by the bootstrap analysis, thus we finally determined

the number of factors to be 6. Based on the base run, constraints were sparingly used on some factors to make them more representative of specific sources. For example, the vanadium concentration in the "secondary formation" factor was pulled down with a dQ of 1%. The constrained run maintained the solution's stability, with mapping rates of all constrained factors to their corresponding base factors being larger than 88%.

PCA was also conducted to identify atmospheric pollution sources. The mass concentrations of 25 chemical species used in PMF were normalized using the following equation:[4]

$$Z_{ij} = \frac{C_{ij} - \overline{C}_j}{\sigma_j} \qquad \text{Eq.S2}$$

where C_{ij} is the mass concentration for the jth species in the ith sample, Z_{ij} is the standardized form of C_{ij}, and $\overline{C}_j$ and σ_j refer to the mean mass concentration and the standard deviation for species j, respectively.

The normalized dataset was then input into the Statistical Product and Service Solutions (SPSS, version 27) software for factor analysis using the Principal Components method. Factors with eigenvalues greater than 1 were extracted, explaining 87.9% of the total variance. A varimax rotation was performed to facilitate interpretation. As shown in Table S4, the marker species for PCA factors are generally consistent with those derived from PMF factors (Table S3), confirming the stability and interpretability of our source apportionment results.

Text S3: Interpretation of PMF source apportionment results

The profiles and time series of the six factors identified from the PMF analysis are presented in Fig. S2 and Fig. S3, respectively. The marker species used to identify their corresponding pollution sources are summarized in Table S3. The peak times for the

mineral dust and BB factors align with the dust storm and the Lag BaOmer events, respectively. The BB factor was characterized by typical wood or straw burning markers such as f_{60}, potassium (K), and WSOC.[5,6] In addition, it exhibited elevated levels of Zn, Cd and As, which also peaked during the Lag BaOmer night (Fig. S1). These heavy metal species co-varied with f_{60}, K and WSOC, confirming their association with the BB event. In samples 0508N1 and 0508N2, the concentration ratios of Zn, Cd, and As were 116:4:1 and 137:7:1, respectively, consistent with charcoal burning particles.[7] In addition, old furniture and processed wood used in the festival bonfires possibly contributed to these emissions. For instance, Zn, commonly found in furniture paint,[8,9] may explain its presence in PM_{10}. The marine factor has high concentrations of Na, Cl and Mg, which are common markers for sea salt particles.[10] The factor with large contributions of V and Ba had elevated concentrations during nighttime easterly winds (Fig. S5) compared to daytime westerly winds from the Mediterranean Sea. Given the proximity of a large cement production facility located approximately 8 km east of the sampling site and the abundance of V and Ba in the raw materials for cement production,[11,12] this factor was attributed to "industrial dust" blown from polluted soils around the local cement factory. The traffic factor was characterized by high Fe, Co, Ni, Cu and Pb, which are important metal species in vehicle exhaust and non-exhaust emissions.[5,13] The traffic factor had relatively low concentrations on May 6th and 7th (Fig. S3), reflecting the lower transportation activity during weekend days. The secondary formation factor increased during the day and decreased at night, suggesting the contribution of photochemical reactions to the oxidation state of particles. Throughout the entire sampling period, the mineral dust contributed approximately half of the PM_{10} loading (Fig. S6b), consistent with long-term observations from filter sampling

conducted between 2019 and 2023 at the same site.[14] A companion paper of this study shows that the dust event on May 5th was originally from the desert area in Saudi Arabia, while the sampling site was also influenced by dust from the Sahara Desert on other sampling days.[15]

There are specific uncertainties in the PMF results. For example, Fe can be a marker species for mineral dust and traffic,[5] while in our case, it is mainly allocated to traffic. In addition to the cement production mentioned above, Ba is also a marker for tire wear particles,[16] while it is low in the profile of the traffic factor. On one hand, the small dataset weakens the representativeness of the PMF source profiles, and caution should be exercised when extrapolating the results to other seasons or regions. On the other hand, the time series of PMF factors are consistent with observed events; with the same dataset, the PCA analysis shows the same source apportionment of those marker species as PMF (Table S4), reinforcing the feasibility of PMF source apportionment to this specific dataset.

Text S4: Linking OP_v^{DTT} and ROS_v of WS-PM_{10} with pollution sources.

OP_v^{DTT} and ROS_v were attributed to different pollution sources with the MLR method. With *i* as sample, *k* as factor, the MLR method attempts to solve the following equation for all the samples:

$$y_i = \sum_{k=1}^{f+1} \beta_k g_{ik} + \varepsilon_i \qquad \text{Eq.S3}$$

where y_i is OP^{DTT} or cellular ROS, β_k is the intrinsic OP or ROS generation potential for each specific pollution source, and g_{ik} is the mass of pollution source derived from PMF analysis. Specifically, $g_{i(f+1)}$ is set to 1, and β_{f+1} is a constant indicating the intercept, which represents the unexplained OP^{DTT} or cellular ROS.[17] ε_i is the residual term accounting for the misfit between observation and model, and the regression seeks to minimize this term.

The SPSS software was used to run the MLR. First, all six factors were input into the MLR, and the Student's *t*-test was used to check if each factor was significantly correlated with the OP^{DTT} or cellular ROS. As a result, mineral dust, industrial dust, BB, and traffic were the four sources that significantly correlated with OP^{DTT}, and their homoscedasticity was confirmed by a Koenker test (null hypothesis: homoscedasticity; $p > 0.05$). Subsequently, an MLR was conducted between these four sources and OP^{DTT}, and the uncertainty of β_k was estimated by bootstrapping 2000 times. On the other side, BB and traffic were the two sources that significantly correlated with cellular ROS. These two sources had significant heteroscedasticity ($p < 0.05$). Therefore, the wild bootstrapping was performed for 2000 times accordingly. In the next step, the contribution of the pollution source k to OP^{DTT} or cellular ROS in sample i was calculated as the product of β_k and g_{ik}. The source apportionment of OP_v^{DTT} and ROS_v to different pollution sources with the PMF-MLR method are as follows:

$$OP_v^{DTT}=(0.190\pm0.114)\cdot C_{Mineral}+(0.230\pm0.052)\cdot C_{Industrial}+(0.324\pm0.128)\cdot C_{BB}+(0.147\pm0.050)\cdot C_{Traffic}+(0.165\pm0.107) \quad \text{Eq.S4}$$

$$ROS_v=(0.580\pm0.054)\cdot C_{BB}+(0.643\pm0.106)\cdot C_{Traffic}+(0.242\pm0.161) \quad \text{Eq.S5}$$

where C indicates the concentration of the PMF factors, the constant before C is the corresponding intrinsic OP or ROS for each factor, and the uncertainty of constants were derived from bootstrapping 2000 times with the SPSS software. The adjusted R^2 for Eq.S4 and Eq.S5 are 0.90 and 0.83, respectively.

Text S5: Parameters used for $OP_{dose,T}$ estimation

The tidal volume and respiratory frequency were set to 5×10^{-4} m^3 and 900 h^{-1}, respectively, representing the condition for a healthy adult at rest.[18] When using the MPPD to estimate the particle deposition rate, the airway morphometry settings included:

Stochastic lung model (60th percentile), a functional residual capacity (FRC) of 3300 mL, an upper respiratory tract (URT) volume of 50 ml. An aerodynamic aerosol classifier (AAC, Cambustion, UK) was used in tandem with SMPS to measure the effective density of particles during the campaign, which was 1.52 g cm^{-3} regardless of particle size. Particle sizes from 1 nm to 10 μm were scanned with 300 intervals. While particle concentrations varied by sampling periods, this did not affect deposition rates under any scenario. The inspiratory fraction was set to 0.5, with no pause fraction. Nasal breathing was simulated, and clearance was not considered. The resulting deposition rates in head airway, tracheobronchial and pulmonary regions are summarized in Table S6.

Text S6: Method to reconstruct OP_v^{DTT} of metals

The OP of single metal species was estimated based on reference values listed in Table S7, in which *x* means the concentration (μmol L^{-1}) of a metal species in the DTT incubation solution and *y* means its expected DTT decay rate (μmol min^{-1}). By considering their antagonistic effects, the summed OP_v^{DTT} of metal species was calculated with the following equation:[19]

$$OP^{DTT}_{metal\ mix}=\sum_{i=1}^{n}[OP_i\cdot\sum_{i\neq j}^{n}(F_{ij}\cdot M_{ij}^{-0.5\cdot\theta_{ij}})] \quad \text{Eq.S6}$$

where *i* and *j* are metal species, M is the interaction factor and an empirical value of 5 was applied.[19] F_{ij} is the exposure factor and θ_{ij} is the geometric/arithmetic mean, which are defined as:

$$F_{ij}=\frac{OP_j}{\sum_{i=1}^{n}OP_i\text{-}OP_i} \quad \text{Eq.S7}$$

$$\theta_{ij}=\frac{\sqrt{OP_i*OP_j}}{(OP_i+OP_j)/2} \quad \text{Eq.S8}$$

Given that copper (Cu) and manganese (Mn) contributed more than 97% of the

OP_v^{DTT} of metal species across all filter samples, metals other than Cu and Mn were grouped together and treated as a single “combined metal species”. This simplification streamlines the calculation by summing the OP contributions of these other metals without individually analyzing their interactions. This approach assumes that the influence of metals other than Cu and Mn is relatively minor and does not significantly alter the overall OP_v^{DTT} results.

Table S1 Basic information of PM_{10} filter samples.

Sample ID	Start time	End time	Sampled air volume (m^3)	PM_{10} concentration ($\mu g\ m^{-3}$)	Anthropogenic-dominant
0502D	May 2, 9:43	May 2, 15:46	410.2	36.8±1.8	yes
0502N*	May 2, 20:28	May 3, 8:52	840.5	33.3±1.7	yes
0503D	May 3, 9:05	May 3, 20:06	746.9	49.3±2.5	no
0503N	May 3, 20:17	May 4, 7:22	751.2	41.9±2.1	yes
0504D	May 4, 7:31	May 4, 20:28	880.1	73.3±3.7	no
0504N*	May 4, 20:36	May 5, 9:06	846.3	79.5±4	no
0505D*	May 5, 9:14	May 5, 20:45	779.5	219.8±11	no
0505N	May 5, 20:55	May 6, 9:11	830.4	68.6±3.4	no
0506D	May 6, 9:19	May 6, 20:33	761.4	35.7±1.8	no
0506N	May 6, 20:41	May 7, 9:11	848.4	33.7±1.7	no
0507D	May 7, 9:16	May 7, 20:47	780.5	21.1±1.1	yes
0507N	May 7, 20:54	May 8, 8:44	801.1	31.1±1.6	no
0508D	May 8, 8:53	May 8, 19:54	745.6	33±1.6	no
0508N1*	May 8, 20:02	May 9, 2:13	418.9	73.3±3.7	yes
0508N2*	May 9, 2:21	May 9, 8:33	419.0	64.2±3.2	yes
0509D1*	May 9, 8:41	May 9, 14:23	386.4	46.6±2.3	no
0509D2*	May 9, 14:29	May 9, 20:31	408.8	26.4±1.3	yes
0509N	May 9, 20:36	May 10, 8:58	838.1	37.9±1.9	yes
0510D	May 10, 9:03	May 10, 20:45	793.1	39.5±2	yes
0510N*	May 10, 20:41	May 11, 8:51	815.7	37.3±1.9	yes

0511D	May 11, 8:59	May 11, 20:02	748.1	36.2±1.8	yes
0514D	May 14, 9:16	May 14, 20:31	761.1	46.2±2.3	no
0514N	May 14, 20:37	May 15, 8:29	805.6	30.4±1.5	yes
0515D	May 15, 8:35	May 15, 21:07	849.5	45.2±2.3	yes
0515N	May 15, 21:13	May 16, 9:47	852.0	50.7±2.5	yes

* The asterisk means that a size-resolved filter set (<0.49 µm, 0.49-0.95 µm, 0.95-1.5 µm, 1.5-3 µm, 3-7.2 µm, 7.2-10 µm) was sampled simultaneously with the PM_{10} sample. Particularly, only one size-resolved filter set was sampled from 8:31-20:42 of May 9th, covering the 0509D1 and 0509D2 bulk PM_{10} samples. The 0505D sample represents the mineral dust event, the 0508N1 and 0508N2 samples represent the BB event. For the remaining four samples, Fig. 1 shows that 0502N and 0510N samples had ⩾50% OP_v^{DTT} contribution from the industrial dust factor and were thus denoted as the "Industry-affected period". The 0504N and 0509D had no single factor that contributed to ⩾40% of OP_v^{DTT} and were denoted as the "Regular period".

Table S2 Cell death rate of A549 after exposure to 0.1 g L^{-1} PM_{10} extract for 24 h.

Sample ID	Cell death rate (%)	Number of tests
0503D	1.7±0.4	6
0503N	3.4±1.4	3
0504D	2.2±0.4	3
0504N	3.4±0.7	4
0505D	2.6±0.8	12
0508N1	2.8±2.2	4
0508N2	4.0±0.9	3
0514D	2.5±0.9	5
0514N	3.2±1.4	4
0515D	2.0±0.7	9
0515N	2.0±0.5	6
Blank	4.2±1.5	10

Table S3 Summary of the characteristic marker species used to identify PMF factors

PMF factors	Marker species
Mineral dust	PM_{10}, Al, Ca
Marine	Na, Mg, Cl
Industrial dust	V, Ba
BB	K, Zn, As, Cd, WSOC, f_{60}
Traffic	Fe, Co, Ni, Cu, Pb
Secondary formation	f_{44}, O_x

Table S4 Loadings of chemical species in the factors resolved by the PCA. Loadings >0.60 are bolded.

	BB	Traffic	Dust	Marine	Industrial dust	Secondary formation
Cd	**0.967**	0.056	-0.094	0.112	-0.006	-0.133
WSOC	**0.936**	0.007	0.138	0.011	0.257	0.006
Zn	**0.927**	0.193	-0.150	0.149	0.046	-0.142
f_{60}	**0.917**	-0.069	-0.065	0.097	-0.062	-0.329
As	**0.908**	0.133	-0.043	0.105	-0.022	-0.206
WSOM	**0.884**	0.053	0.171	-0.046	0.308	0.196
K	**0.842**	0.025	0.267	0.389	0.144	-0.100
Mn	**0.711**	0.566	0.131	0.178	-0.099	0.148
Fe	-0.074	**0.942**	0.177	0.016	-0.036	0.011
Co	0.198	**0.820**	-0.289	0.155	-0.056	0.064
Cu	-0.030	**0.783**	-0.049	-0.458	0.072	0.013
Ni	0.087	**0.663**	-0.167	-0.020	-0.296	-0.577
Pb	0.459	**0.605**	-0.050	-0.152	0.083	-0.062
SO_4	0.186	0.595	0.031	0.414	0.331	-0.161
NO_3	0.336	0.480	0.130	0.425	0.399	-0.383
PM10	0.189	-0.164	**0.935**	0.137	0.106	0.021
Ca	0.013	-0.187	**0.903**	-0.096	0.231	0.086
Al	-0.020	0.278	**0.864**	0.246	-0.021	-0.088
Mg	0.112	0.033	0.200	**0.928**	-0.101	0.007
Chl	0.494	-0.210	-0.077	**0.790**	-0.079	0.067
Ba	0.290	-0.129	0.051	-0.084	**0.842**	-0.199
V	-0.141	0.450	0.292	-0.057	**0.707**	0.067
Na	-0.198	0.485	-0.022	0.492	-0.551	-0.060
f_{44}	-0.525	0.006	-0.118	0.151	-0.211	**0.659**
O_x	-0.193	0.067	0.538	-0.209	-0.352	0.567
Variance (%)	34.9	17.3	13.7	11.0	6.7	4.3
Accumulated Variance (%)	34.9	52.2	65.9	76.9	83.6	87.9

Table S5 PM concentration and OP^{DTT} of WS components in different sampling periods. Literature values are provided as comparison.

Site	Sampling period	PM size	PM concentration ($\mu g\ m^{-3}$)	OP_m^{DTT} ($pmol\ min^{-1}\ \mu g^{-1}$)	OP_v^{DTT} ($nmol\ min^{-1}\ m^{-3}$)	Reference
Rehovot, Israel	BB	PM_{10}	68.7±6.4	39.9±12.4	2.78±1.11	This study
Rehovot, Israel	Dust	PM_{10}	219.8±11.0	6.2±0.4	1.36±0.09	This study
Rehovot, Israel	Non-event	PM_{10}	42.5±14.8	20.9±7.2	0.88±0.37	This study
Los Angeles, USA	BB	$PM_{2.5}$	/	14-25	0.4-0.8	Verma et al., 2009[20]
Patiala, India	BB	$PM_{2.5}$	271±122	14±5.1	55±12	Patel et al., 2021[21]
Amazon, Brazil	BB	PM_{10}	/	61±7	/	Tuet et al., 2019[22]
Laboratory	BB	$PM_{2.1}$	/	9-30	/	Fushimi et al., 2017[23]
Laboratory	BB	$PM_{2.5}$	/	12.5-20.6	/	Fan et al., 2018[24]
Lecce, Italy	Dust	PM_{10}	137.6±93.9	3.1±1.7	0.32±0.11	Chirizzi et al., 2017[25]
Tehren (Rural), Iran	Dust	PM_{10}	140±54	21.3±10.7	4.04±2.63	Rezaei et al., 2018[26]
Fukuoka, Japan	Dust	$PM_{2.5-10}$	36.0±18.5	21.1±5.0	0.73±0.29	Nishita-Hara et al., 2019[27]
Riyadh, Saudi Arabia	Dust	PM_{10}	218.2±34.8	9.3±0.9	1.50±0.21	Altuwayjiri et al., 2022[4]
Xianghe, China	Dust	$PM_{2.5}$	548	2.7	1.5	Cheung et al., 2024[28]

Table S6 Regional deposition efficiency in the human respiratory tract as predicted from the MPPD model for particle in different size ranges.

	Head	Tracheobronchial	Pulmonary	Total
<0.49 μm	0.226	0.216	0.102	0.545
0.49-0.95	0.064	0.050	0.059	0.173
0.95-1.5	0.156	0.055	0.093	0.303
1.5-3	0.366	0.066	0.132	0.564
3-7.2	0.754	0.061	0.072	0.887
7.2-10	0.953	0.028	0.006	0.987

Table S7 Regression equations for the DTT decay rate of individual metal species. *x* means the concentration (μmol L^{-1}) of a metal species in the DTT incubation solution and *y* means its expected DTT decay rate (μmol min^{-1}).

Metal species	Regression Equation	Reference
Cu	$y = 0.608x^{0.3}$	Expósito et al., 2024[29]
Mn	$y = 0.257x^{0.3}$	Expósito et al., 2024[29]
Co	$y = 0.27x$	Charrier and Anastasio, 2012[30]
Ni	$y = 0.106x$	Charrier and Anastasio, 2012[30]
V	$y = 0.101x$	Charrier and Anastasio, 2012[30]
Pb	$y = 0.064x$	Charrier and Anastasio, 2012[30]
Fe	$y = 0.035x$	Charrier and Anastasio, 2012[30]

Table S8 The influence of Chelex treatment to 2-methoxy-1.4-hydroquinone or 1,4-naphthaquinone.

Base solution[a]	Treatment	DTT consumption rate ($\mu M\ min^{-1}$)
1 mg L^{-1} MHQ	Not treated	0.15±0.01
1 mg L^{-1} MHQ	Shaking for 12 h without Chelex	0.14±0.01
1 mg L^{-1} MHQ	Shaking for 12 h with Chelex	0.16±0.02
0.15 mg L^{-1} 1,4-NQ	Not treated	0.52±0.03
0.15 mg L^{-1} 1,4-NQ	Shaking for 12 h without Chelex	0.53±0.05
0.15 mg L^{-1} 1,4-NQ	Shaking for 12 h with Chelex	0.53±0.05

[a] MHQ: 2-methoxy-1.4-hydroquinone; 1,4-NQ: 1,4-naphthaquinone. The chosen concentrations of organics ensured that less than 25% of the initial DTT was consumed after 40 min of reaction.

Fig. S1 The time series of abundant species in the BB factor from the PMF analysis: (a) f_{60}, (b) K, (c) WSOC, (d) Zn, (e) Cd, (f) As. The uncertainty was depicted as shadow.

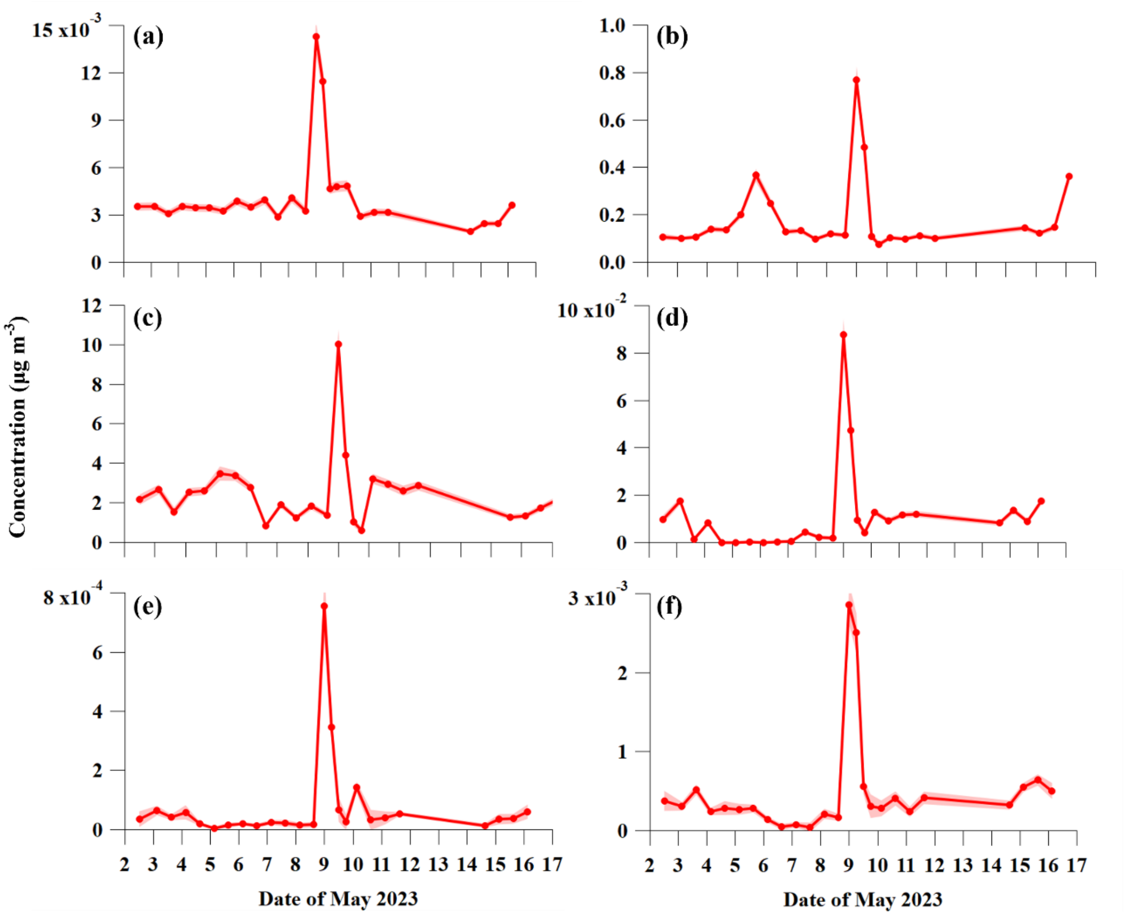

Fig. S2 The profile of factors derived from the PMF analysis: (a) mineral dust, (b) marine, (c) industrial dust, (d) biomass burning, (e) traffic, (f) secondary formation. The green column with the left axis denotes the concentration of species; the orange square marker with the right axis indicates the factor contribution to species.

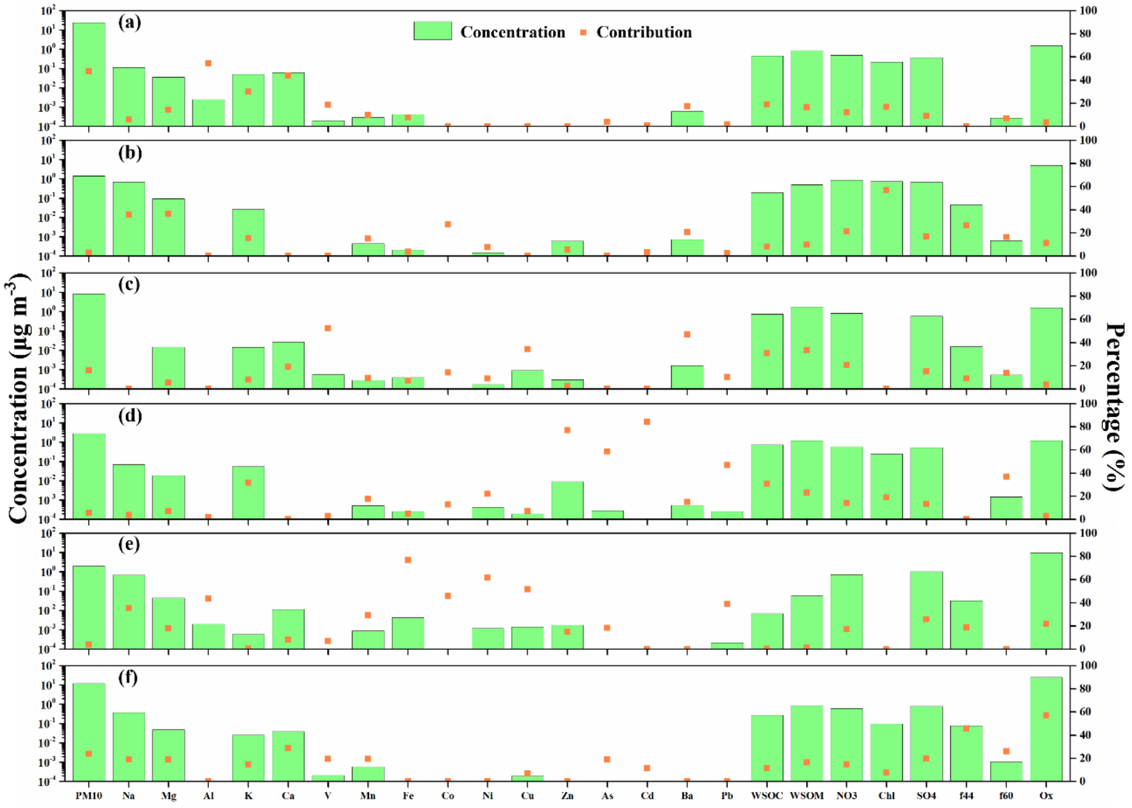

Fig. S3 The time series of factor contribution derived from the PMF analysis: (a) mineral dust, (b) marine, (c) industrial dust, (d) biomass burning, (e) traffic, (f) secondary formation. For each factor, its contribution is normalized by its average contribution across the entire period.

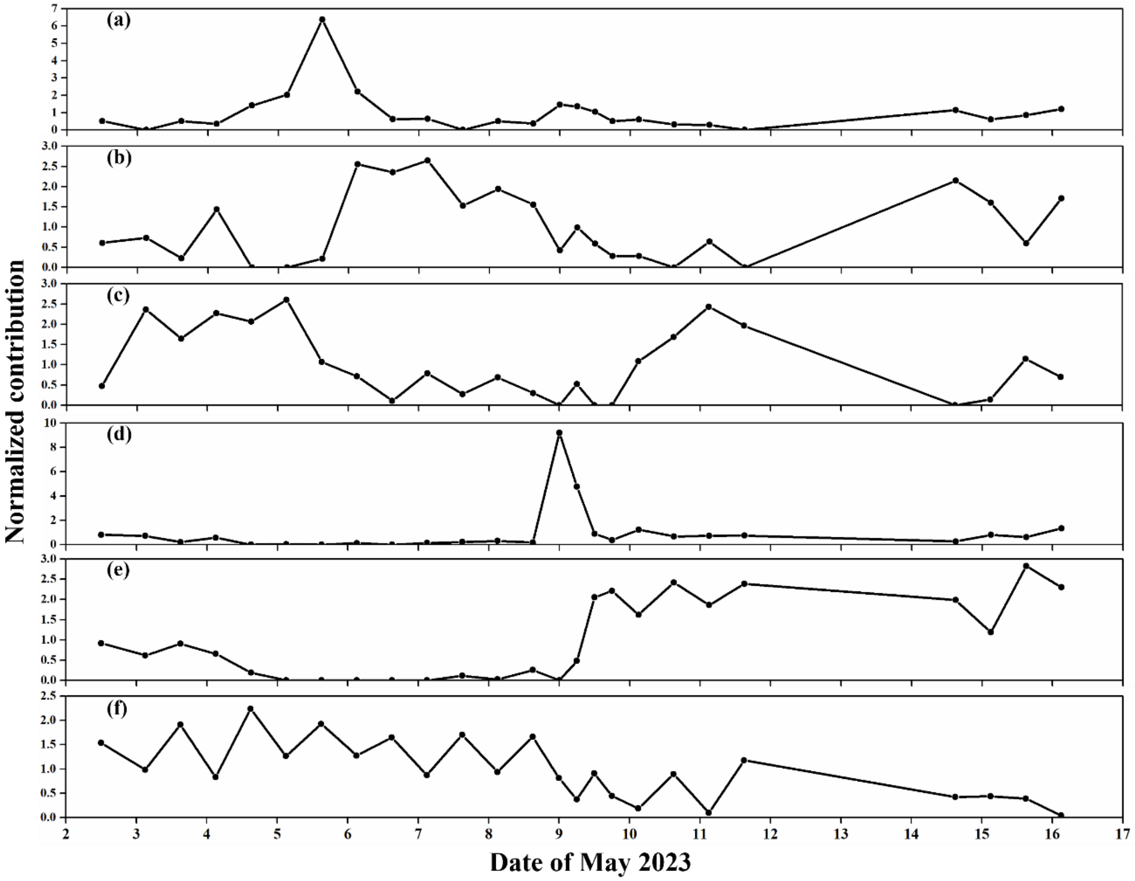

Fig. S4 The IM and IS when different factor numbers are used in the PMF model.

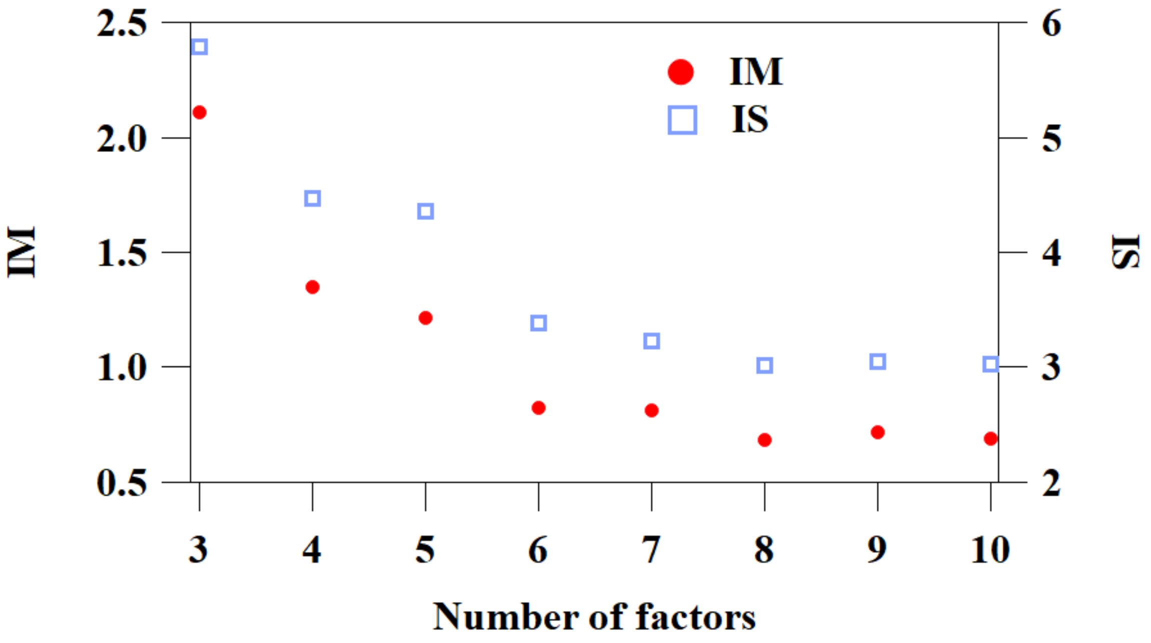

Fig. S5 The wind rose for each filter sampling period.

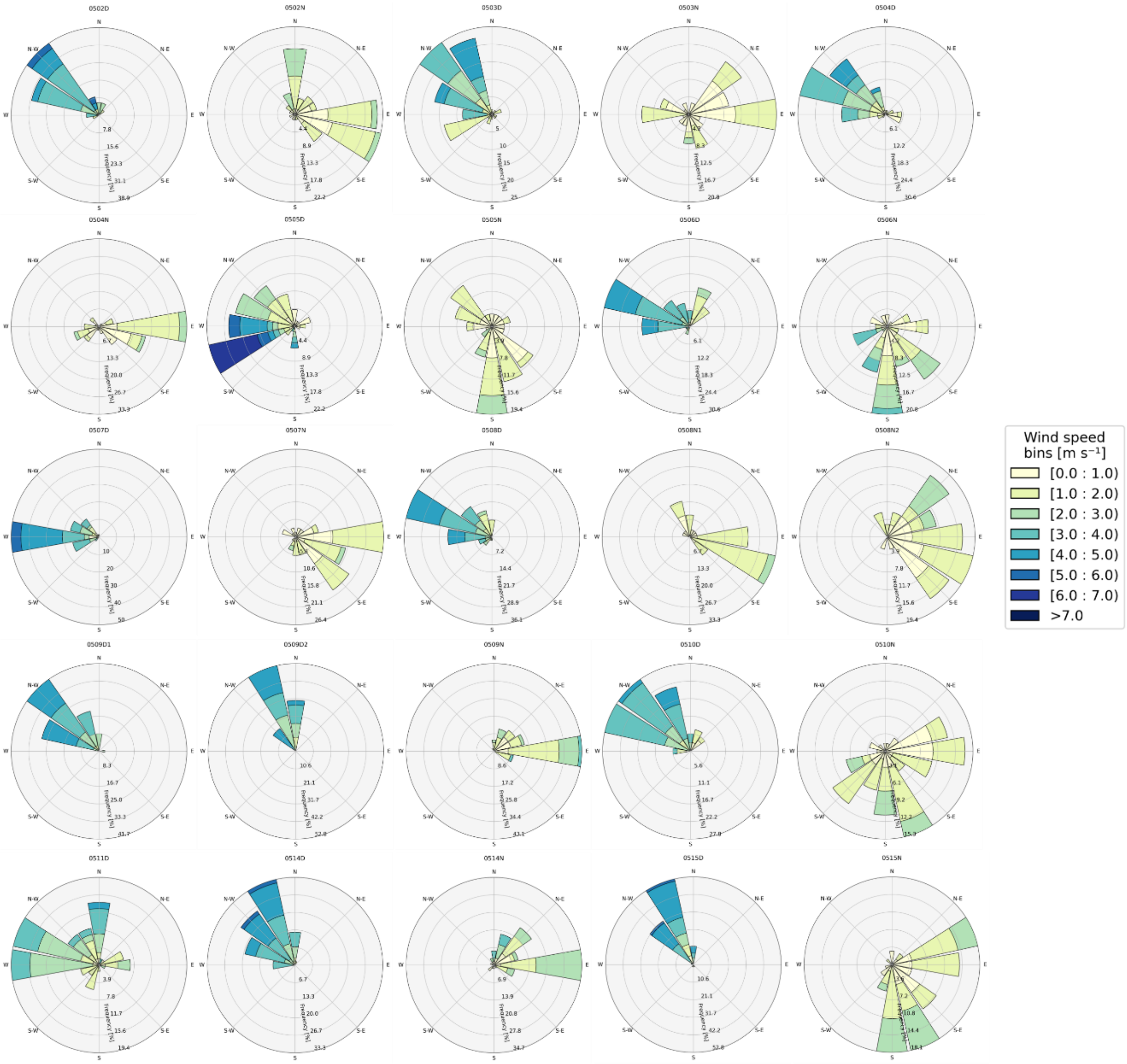

Fig. S6 (a) The mass ratio of PMF factors for each filter sampling period; (b) The contribution of PMF factors to PM_{10} during the entire sampling period.

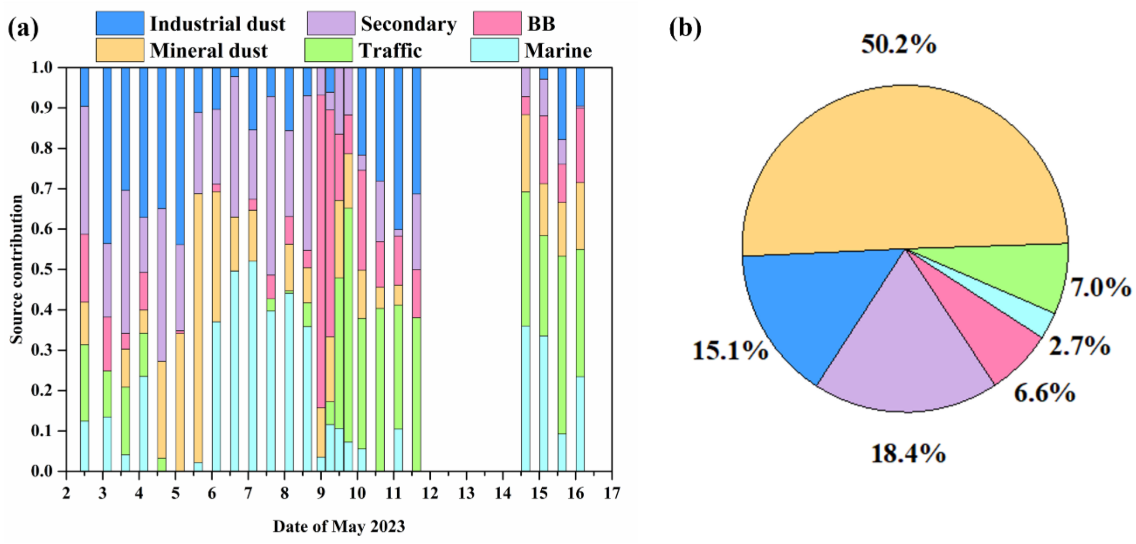

Fig. S7 OP_m^{DTT} and OP_v^{DTT} for size-resolved fractions of WS-PM: (a) Regular period; (b) BB event; (c) Mineral dust event, and (d) Industry-affected period. The proportion (in %) of OP_v^{DTT} for each size bin in the summed OP_v^{DTT} is labelled on the top of each column.

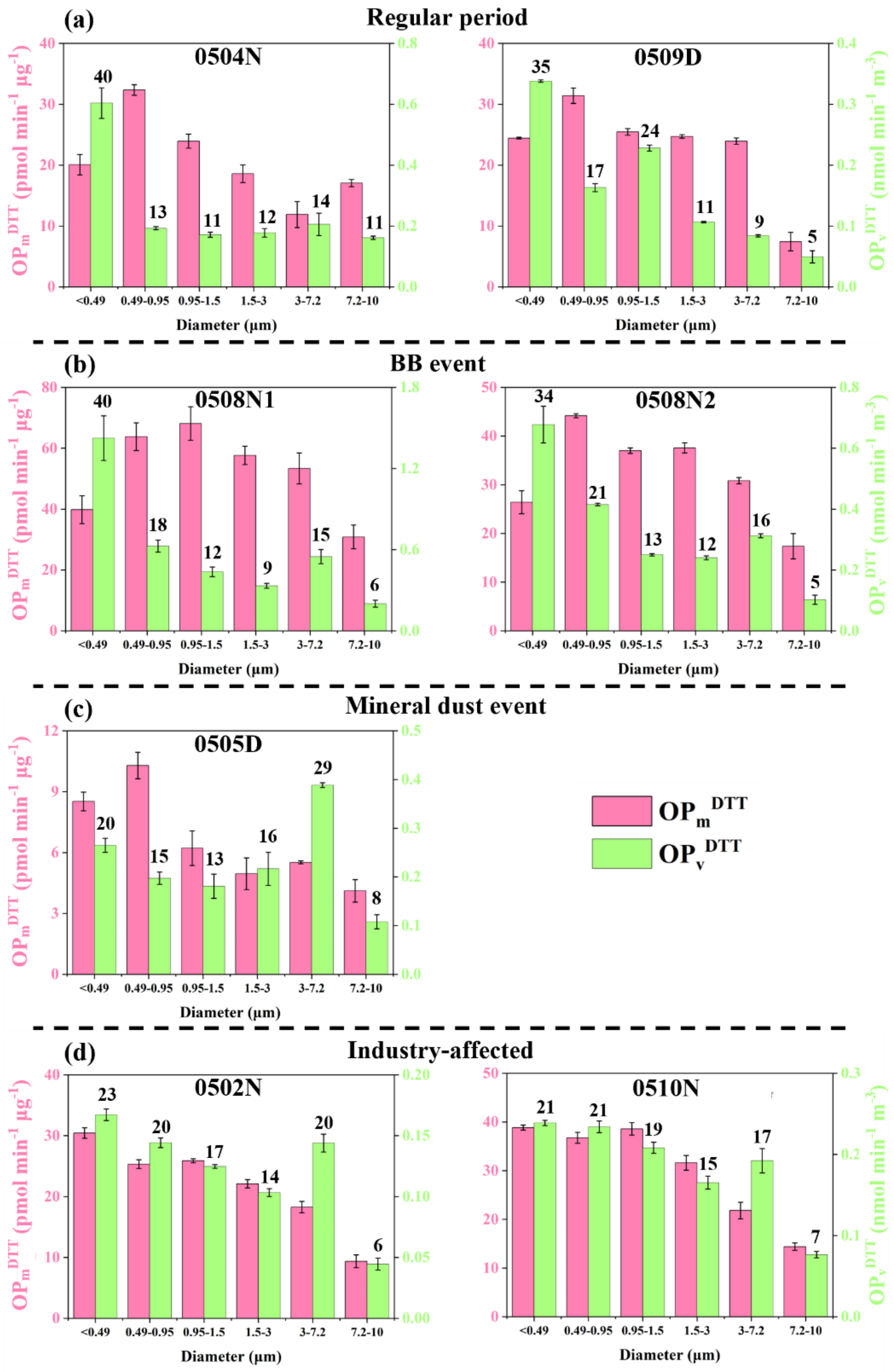

Fig. S8 Size-dependent $OP_{dose,T}$ in the entire human respiratory tract for different periods: (a) Regular period, (b) BB event, (c) Mineral dust event; and (d) Industry-affected period.

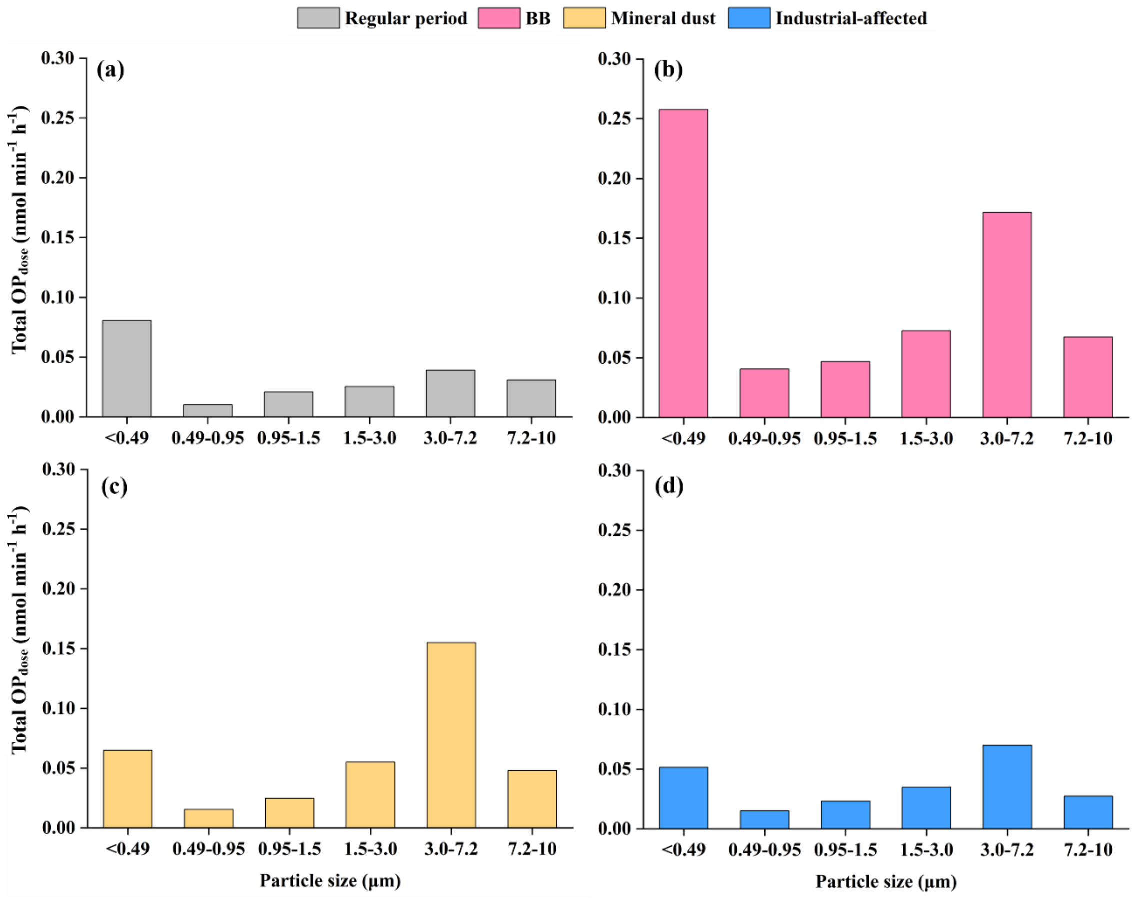

Fig. S9 Size-dependent $OP_{dose,T}$ in the human pulmonary region for different periods: (a) Regular period, (b) BB event, (c) Mineral dust event; and (d) Industry-affected period.

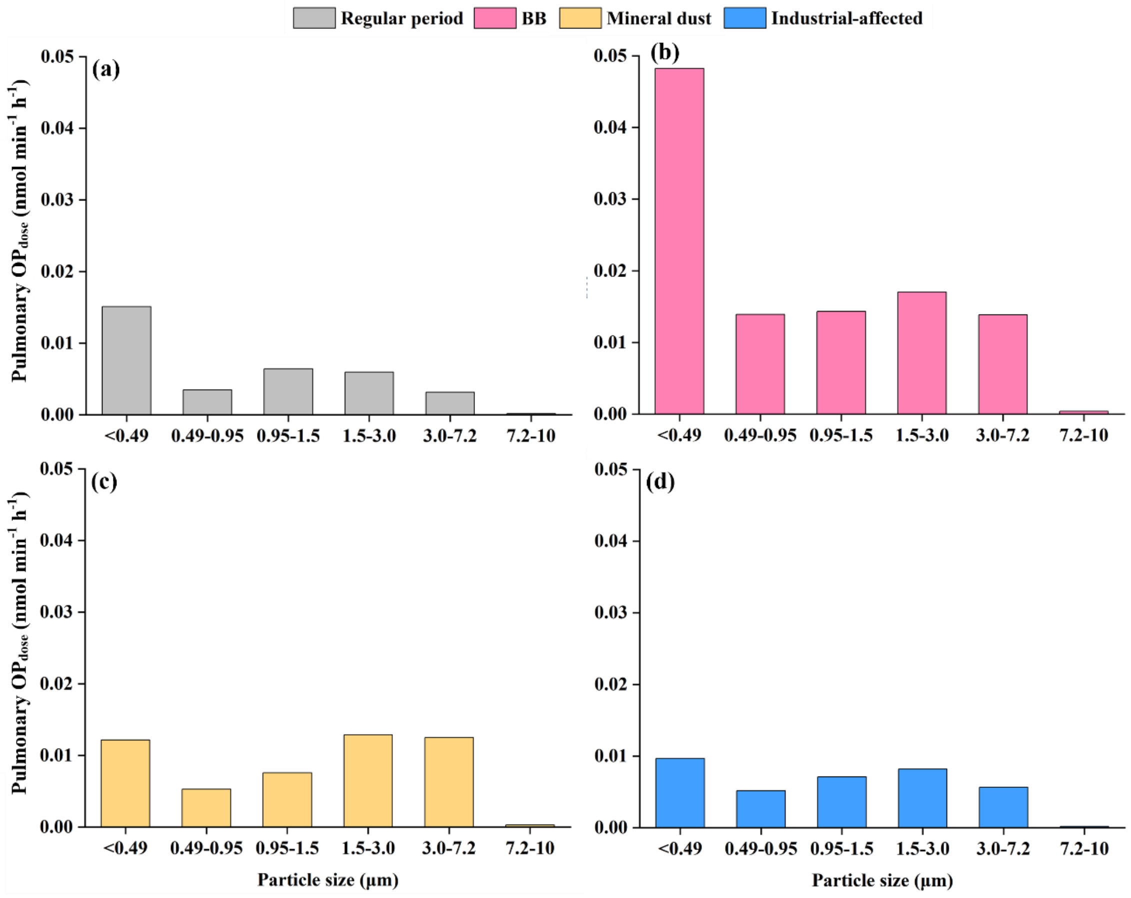

Fig. S10 Size-dependent $OP_{dose,T}$ in the human tracheobronchial region for different periods: (a) Regular period, (b) BB event, (c) Mineral dust event; and (d) Industry-affected period.

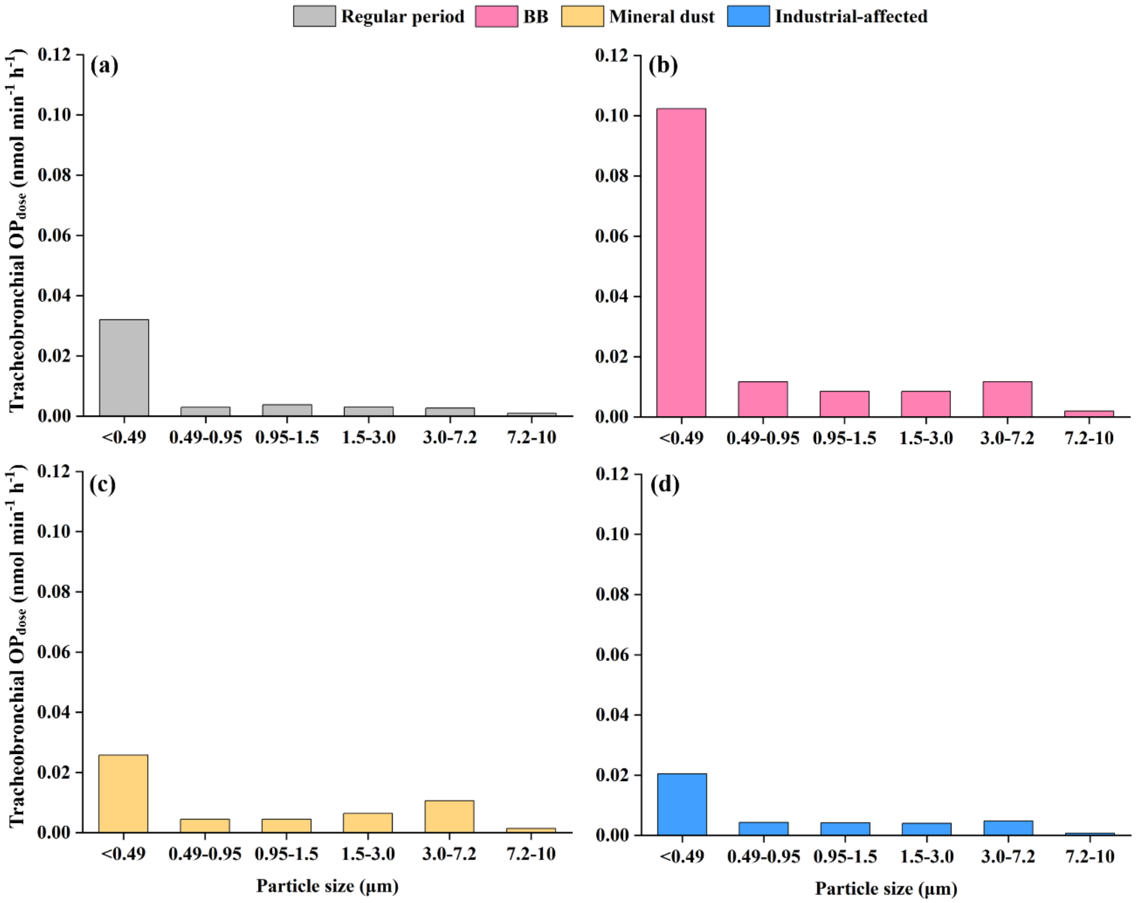

Fig. S11 Size-dependent $OP_{dose,T}$ in the human head airways for different periods: (a) Regular period, (b) BB event, (c) Mineral dust event; and (d) Industry-affected period.

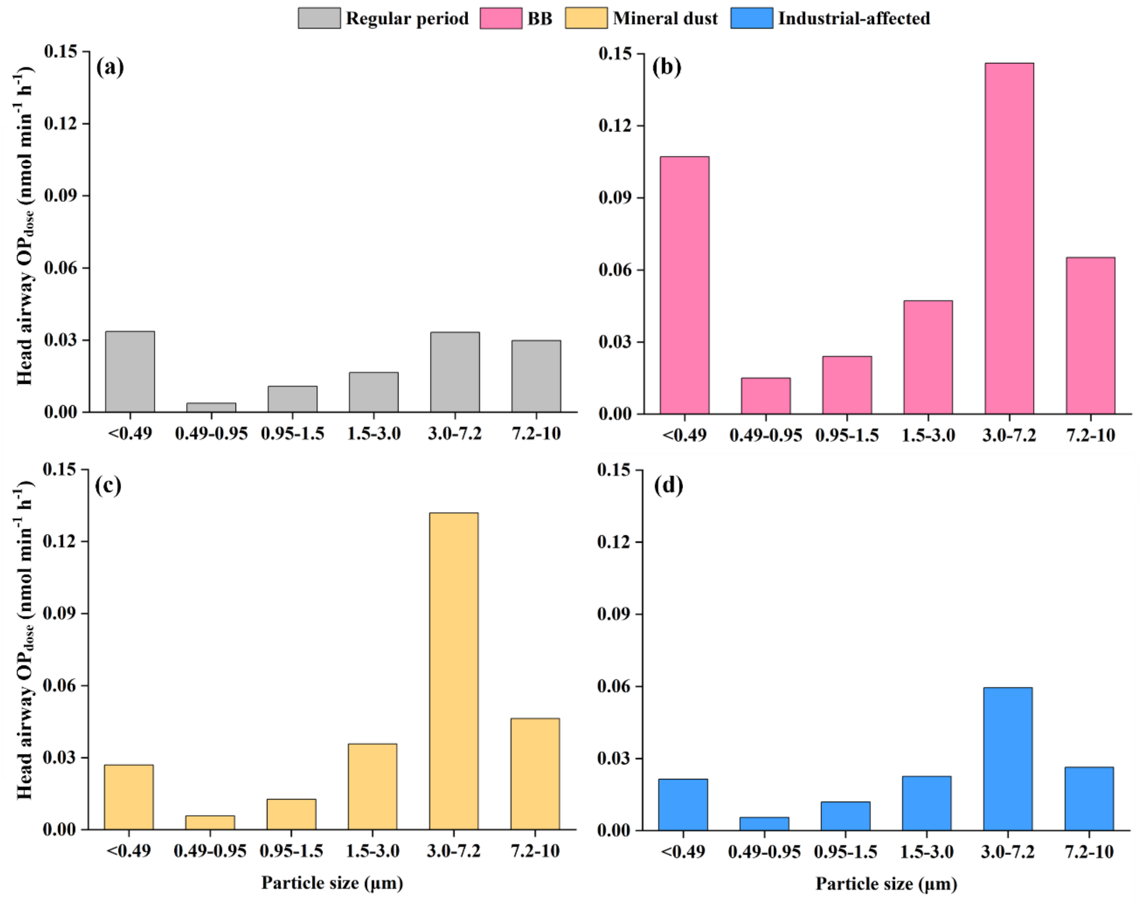

References

(1) Fang, T.; Verma, V.; Bates, J. T.; Abrams, J.; Klein, M.; Strickland, M. J.; Sarnat, S. E.; Chang, H. H.; Mulholland, J. A.; Tolbert, P. E.; Russell, A. G.; Weber, R. J. Oxidative Potential of Ambient Water-Soluble PM2.5 in the Southeastern United States: Contrasts in Sources and Health Associations between Ascorbic Acid (AA) and Dithiothreitol (DTT) Assays. Atmos. Chem. Phys. 2016, 16 (6), 3865–3879. https://doi.org/10.5194/acp-16-3865-2016.

(2) Grange, S. K.; Fischer, A.; Zellweger, C.; Alastuey, A.; Querol, X.; Jaffrezo, J.-L.; Weber, S.; Uzu, G.; Hueglin, C. Switzerland's PM_{10} and $PM_{2.5}$ Environmental Increments Show the Importance of Non-Exhaust Emissions. Atmos. Environ. X 2021, 12, 100145. https://doi.org/10.1016/j.aeaoa.2021.100145.

(3) Harrison, R. M.; Beddows, D. C. S.; Dall'Osto, M. PMF Analysis of Wide-Range Particle Size Spectra Collected on a Major Highway. Environ. Sci. Technol. 2011, 45 (13), 5522–5528. https://doi.org/10.1021/es2006622.

(4) Altuwayjiri, A.; Pirhadi, M.; Kalafy, M.; Alharbi, B.; Sioutas, C. Impact of Different Sources on the Oxidative Potential of Ambient Particulate Matter PM_{10} in Riyadh, Saudi Arabia: A Focus on Dust Emissions. Sci. Total Environ. 2022, 806, 150590. https://doi.org/10.1016/j.scitotenv.2021.150590.

(5) Weber, S.; Salameh, D.; Albinet, A.; Alleman, L. Y.; Waked, A.; Besombes, J.-L.; Jacob, V.; Guillaud, G.; Meshbah, B.; Rocq, B.; Hulin, A.; Dominik-Sègue, M.; Chrétien, E.; Jaffrezo, J.-L.; Favez, O. Comparison of PM_{10} Sources Profiles at 15 French Sites Using a Harmonized Constrained Positive Matrix Factorization Approach. Atmosphere (Basel). 2019, 10 (6), 310.

https://doi.org/10.3390/atmos10060310.

(6) Fang, Z.; Deng, W.; Zhang, Y.; Ding, X.; Tang, M.; Liu, T.; Hu, Q.; Zhu, M.; Wang, Z.; Yang, W.; Huang, Z.; Song, W.; Bi, X.; Chen, J.; Sun, Y.; George, C.; Wang, X. Open Burning of Rice, Corn and Wheat Straws: Primary Emissions, Photochemical Aging, and Secondary Organic Aerosol Formation. Atmos. Chem. Phys. 2017, 17 (24), 14821–14839. https://doi.org/10.5194/acp-17-14821-2017.

(7) Susaya, J.; Kim, K.-H.; Ahn, J.-W.; Jung, M.-C.; Kang, C.-H. BBQ Charcoal Combustion as an Important Source of Trace Metal Exposure to Humans. J. Hazard. Mater. 2010, 176 (1), 932–937. https://doi.org/10.1016/j.jhazmat.2009.11.129.

(8) Huang, S. L.; Yin, C.-Y.; Yap, S. Y. Particle Size and Metals Concentrations of Dust from a Paint Manufacturing Plant. J. Hazard. Mater. 2010, 174 (1), 839–842. https://doi.org/10.1016/j.jhazmat.2009.09.129.

(9) Megertu, D. G.; Bayissa, L. D. Heavy Metal Contents of Selected Commercially Available Oil-Based House Paints Intended for Residential Use in Ethiopia. Environ. Sci. Pollut. Res. 2020, 27 (14), 17175–17183. https://doi.org/10.1007/s11356-020-08297-z.

(10) White, W. H. Chemical Markers for Sea Salt in IMPROVE Aerosol Data. Atmos. Environ. 2008, 42 (2), 261–274. https://doi.org/10.1016/j.atmosenv.2007.09.040.

(11) Vollpracht, A.; Brameshuber, W. Binding and Leaching of Trace Elements in Portland Cement Pastes. Cem. Concr. Res. 2016, 79, 76–92. https://doi.org/10.1016/j.cemconres.2015.08.002.

(12) Saidi, I.; Ben Abdelmalek, J.; Ben Said, O.; Chicharo, L.; Beyrem, H. Chemical Composition and Heavy Metal Content of Portland Cement in Northern Tunisia.

Iran. J. Chem. Chem. Eng. 2020, 39 (3), 147–158. https://doi.org/10.30492/ijcce.2020.34434.

(13) Charron, A.; Polo-Rehn, L.; Besombes, J. L.; Golly, B.; Buisson, C.; Chanut, H.; Marchand, N.; Guillaud, G.; Jaffrezo, J. L. Identification and Quantification of Particulate Tracers of Exhaust and Non-Exhaust Vehicle Emissions. Atmos. Chem. Phys. 2019, 19 (7), 5187–5207. https://doi.org/10.5194/acp-19-5187-2019.

(14) Liu, X.; Turner, J. R.; Oxford, C. R.; McNeill, J.; Walsh, B.; Le Roy, E.; Weagle, C. L.; Stone, E.; Zhu, H.; Liu, W.; Wei, Z.; Hyslop, N. P.; Giacomo, J.; Dillner, A. M.; Salam, A.; Hossen, A.; Islam, Z.; Abboud, I.; Akoshile, C.; Amador-Muñoz, O.; Anh, N. X.; Asfaw, A.; Balasubramanian, R.; Chang, R. Y.-W.; Coburn, C.; Dey, S.; Diner, D. J.; Dong, J.; Farrah, T.; Gahungu, P.; Garland, R. M.; Grutter de la Mora, M.; Hasheminassab, S.; John, J.; Kim, J.; Kim, J. S.; Langerman, K.; Lee, P.-C.; Lestari, P.; Liu, Y.; Mamo, T.; Martins, M.; Mayol-Bracero, O. L.; Naidoo, M.; Park, S. S.; Schechner, Y.; Schofield, R.; Tripathi, S. N.; Windwer, E.; Wu, M.-T.; Zhang, Q.; Brauer, M.; Rudich, Y.; Martin, R. V. Elemental Characterization of Ambient Particulate Matter for a Globally Distributed Monitoring Network: Methodology and Implications. ACS ES&T Air 2024, 1 (4), 283–293. https://doi.org/10.1021/acsestair.3c00069.

(15) Ajith, T. C.; Windwer, E.; Li, C.; Fang, Z.; Kompalli, S. K.; Nursanto, F. R.; Olayemi, T. E.; Ese, J. I.; Sharpe, S. A. L.; Fraund, M.; Moffet, R. C.; Laskin, A.; Fry, J. L.; Rudich, Y. Investigating New Particle Formation and Growth Over an Urban Location in the Eastern Mediterranean. J. Geophys. Res. Atmos. 2024, 129 (23), e2024JD041802. https://doi.org/10.1029/2024JD041802.

(16) Lopez, B.; Wang, X.; Chen, L.-W. A.; Ma, T.; Mendez-Jimenez, D.; Cobb, L. C.; Frederickson, C.; Fang, T.; Hwang, B.; Shiraiwa, M.; Park, M.; Park, K.; Yao, Q.; Yoon, S.; Jung, H. Metal Contents and Size Distributions of Brake and Tire Wear Particles Dispersed in the Near-Road Environment. Sci. Total Environ. 2023, 883, 163561. https://doi.org/10.1016/j.scitotenv.2023.163561.

(17) Weber, S.; Uzu, G.; Favez, O.; Borlaza, L. J. S.; Calas, A.; Salameh, D.; Chevrier, F.; Allard, J.; Besombes, J. L.; Albinet, A.; Pontet, S.; Mesbah, B.; Gille, G.; Zhang, S.; Pallares, C.; Leoz-Garziandia, E.; Jaffrezo, J. L. Source Apportionment of Atmospheric PM10 Oxidative Potential: Synthesis of 15 Year-Round Urban Datasets in France. Atmos. Chem. Phys. 2021, 21 (14), 11353–11378. https://doi.org/10.5194/acp-21-11353-2021.

(18) Hulbert, D. Get Through MCEM Part A: MCQs. CRC Press, 2009.

(19) Guo, H.; Fu, H.; Jin, L.; Huang, S.; Li, X. Quantification of Synergistic, Additive and Antagonistic Effects of Aerosol Components on Total Oxidative Potential. Chemosphere 2020, 252, 126573. https://doi.org/10.1016/j.chemosphere.2020.126573.

(20) Verma, V.; Polidori, A.; Schauer, J. J.; Shafer, M. M.; Cassee, F. R.; Sioutas, C. Physicochemical and Toxicological Profiles of Particulate Matter in Los Angeles during the October 2007 Southern California Wildfires. Environ. Sci. Technol. 2009, 43 (3), 954–960. https://doi.org/10.1021/es8021667.

(21) Patel, A.; Satish, R.; Rastogi, N. Remarkably High Oxidative Potential of Atmospheric $PM_{2.5}$ Coming from a Large-Scale Paddy-Residue Burning over the Northwestern Indo-Gangetic Plain. Acs Earth Space Chem. 2021.

https://doi.org/10.1021/acsearthspacechem.1c00125.

(22) Tuet, W. Y.; Liu, F.; de Oliveira Alves, N.; Fok, S.; Artaxo, P.; Vasconcellos, P.; Champion, J. A.; Ng, N. L. Chemical Oxidative Potential and Cellular Oxidative Stress from Open Biomass Burning Aerosol. Environ. Sci. Technol. Lett. 2019, 6 (3), 126–132. https://doi.org/10.1021/acs.estlett.9b00060.

(23) Fushimi, A.; Saitoh, K.; Hayashi, K.; Ono, K.; Fujitani, Y.; Villalobos, A. M.; Shelton, B. R.; Takami, A.; Tanabe, K.; Schauer, J. J. Chemical Characterization and Oxidative Potential of Particles Emitted from Open Burning of Cereal Straws and Rice Husk under Flaming and Smoldering Conditions. Atmos. Environ. 2017, 163, 118–127. https://doi.org/10.1016/j.atmosenv.2017.05.037.

(24) Fan, X.; Li, M.; Cao, T.; Cheng, C.; Li, F.; Xie, Y.; Wei, S.; Song, J.; Peng, P. Optical Properties and Oxidative Potential of Water- and Alkaline-Soluble Brown Carbon in Smoke Particles Emitted from Laboratory Simulated Biomass Burning. Atmos. Environ. 2018, 194, 48–57. https://doi.org/10.1016/j.atmosenv.2018.09.025.

(25) Chirizzi, D.; Cesari, D.; Guascito, M. R.; Dinoi, A.; Giotta, L.; Donateo, A.; Contini, D. Influence of Saharan Dust Outbreaks and Carbon Content on Oxidative Potential of Water-Soluble Fractions of $PM_{2.5}$ and PM_{10}. Atmos. Environ. 2017, 163, 1–8. https://doi.org/10.1016/j.atmosenv.2017.05.021.

(26) Rezaei, S.; Naddafi, K.; Hassanvand, M. S.; Nabizadeh, R.; Yunesian, M.; Ghanbarian, M.; Atafar, Z.; Faraji, M.; Nazmara, S.; Mahmoudi, B.; Ghozikali, M. G.; ghanbarian, M.; Gholampour, A. Physiochemical Characteristics and Oxidative Potential of Ambient Air Particulate Matter (PM_{10}) during Dust and Non-Dust Storm Events: A Case Study in Tehran, Iran. J. Environ. Heal. Sci. Eng. 2018, 16

(2), 147–158. https://doi.org/10.1007/s40201-018-0303-9.

(27) Nishita-Hara, C.; Hirabayashi, M.; Hara, K.; Yamazaki, A.; Hayashi, M. Dithiothreitol-Measured Oxidative Potential of Size-Segregated Particulate Matter in Fukuoka, Japan: Effects of Asian Dust Events. GeoHealth 2019, 3 (6), 160–173. https://doi.org/10.1029/2019GH000189.

(28) Cheung, R. K. Y.; Zhang, J.; Wang, T.; Kattner, L.; Bogler, S.; Puthussery, J. V; Huang, R.-J.; Gysel-Beer, M.; Slowik, J. G.; Verma, V.; Prevot, A. S. H.; El Haddad, I.; Bell, D. M.; Modini, R. L. Online Measurements during Simulated Atmospheric Aging Track the Strongly Increasing Oxidative Potential of Complex Combustion Aerosols Relative to Their Primary Emissions. Environ. Sci. Technol. Lett. 2024. https://doi.org/10.1021/acs.estlett.4c00956.

(29) Expósito, A.; Maillo, J.; Uriarte, I.; Santibáñez, M.; Fernández-Olmo, I. Kinetics of Ascorbate and Dithiothreitol Oxidation by Soluble Copper, Iron, and Manganese, and 1,4-Naphthoquinone: Influence of the Species Concentration and the Type of Fluid. Chemosphere 2024, 361, 142435. https://doi.org/10.1016/j.chemosphere.2024.142435.

(30) Charrier, J. G.; Anastasio, C. On Dithiothreitol (DTT) as a Measure of Oxidative Potential for Ambient Particles: Evidence for the Importance of Soluble Transition Metals. Atmos. Chem. Phys. 2012, 12 (19), 9321–9333. https://doi.org/10.5194/acp-12-9321-2012.
